# Comparative Efficacy of Phacotrabeculectomy versus Trabeculectomy with or without Later Phacoemulsification: A Systematic Review with Meta-Analyses

**DOI:** 10.1155/2021/6682534

**Published:** 2021-02-13

**Authors:** Afrouz Ahmadzadeh, Line Kessel, Yousif Subhi, Daniella Bach-Holm

**Affiliations:** ^1^Department of Ophthalmology, Rigshospitalet-Glostrup, Copenhagen, Denmark; ^2^Department of Clinical Medicine, Faculty of Health Sciences, University of Copenhagen, Copenhagen, Denmark

## Abstract

There is no consensus on the surgical management of coexisting cataract in patients who undergo glaucoma surgery. In this study, we systematically reviewed the literature to compare the efficacy and safety of phacotrabeculectomy and trabeculectomy either alone or followed by later phacoemulsification. We systematically searched the literature databases PubMed/MEDLINE, EMBASE, and the Cochrane Central. Eligible studies were comparative trials of eyes with glaucoma that underwent either phacotrabeculectomy or trabeculectomy with or without later phacoemulsification. Our primary outcome measure was intraocular pressure (IOP) control closest to 12 months. Secondary outcome measures were efficacy closest to 12 months in terms of visual acuity, visual field, prevalence of complications, needling or revision, number of antiglaucomatous medications, and surgical success. We identified 25 studies with a total of 4,749 eyes. The IOP did not differ significantly between those who underwent phacotrabeculectomy versus trabeculectomy with (MD: 0.63, CI95%: −0.32, 1.59, *p*=0.19) or without later phacoemulsification (MD: −0.52, CI95%: −1.45, 0.40, *p*=0.27). However, phacotrabeculectomy was associated with lower risk of complications (RR: 0.80, CI95%: 0.67, 0.95, *p*=0.01) and better visual acuity corresponding to a 1.4-line difference (MD: −0.14, CI95%: −0.27, −0.95, *p*=0.03) compared to trabeculectomy. Other secondary outcome measures did not differ significantly (visual field, needling or revision, number of antiglaucomatous medications, and surgical success). In conclusion, postoperative IOP is comparable, and the number of complications is lower when phacotrabeculectomy is compared to trabeculectomy with or without later phacoemulsification in patients with coexisting glaucoma and cataract. However, our study also reveals that the level of evidence is low, and randomized clinical trials are warranted.

## 1. Introduction

Cataract and glaucoma are globally the most common causes of blindness and they frequently coexist [1–3]. It is believed that up to 10% of the elderly with cataracts have ocular hypertension (OHT) or glaucoma [4, 5], and in 2040, glaucoma is estimated to affect 111.8 million individuals worldwide [6]. Elevated intraocular pressure (IOP) is the only modifiable risk factor for the progression of visual field loss in patients with glaucoma. Among those who cannot achieve satisfactory target IOP and preservation of visual function, the current best practice is to consider filtration surgery. The most widely performed IOP-lowering procedure worldwide is trabeculectomy whereby a channel between the anterior chamber of the eye and the subconjunctival space is created [7].

An important number of patients requiring surgical intervention for glaucoma present with coexisting cataract, and it remains debated how best to manage these patients. Prior to trabeculectomy, it may be tempting to remove the lens and replace it with a thinner intraocular lens to increase anterior chamber depth in order to reduce the risk of the postoperative shallow anterior chamber [8]. However, trabeculectomy is often performed prior to cataract surgery since the optic nerve head in these patients is at high risk of damage from postoperative IOP spikes, which is a known phenomenon after cataract surgery [9], and also because postponing the trabeculectomy may increase the risk of visual field loss. On the other hand, performing a trabeculectomy in a phakic eye is challenging due to vitreous pressure that pushes the phakic lens forward during the operation. Further, trabeculectomy may advance cataract progression, and 6–58 % of the patients have been reported to convert from no cataract at the time of filtration surgery to cataract requiring surgery within the first year [10–12]. Trabeculectomy-induced cataract progression which necessitates cataract surgery may lead to a subsequent increase in IOP due to bleb failure [13, 14]. It is believed that bleb failure is related to postoperative inflammation and a change in the microenvironment, causing the closure of the filtration route of the aqueous humor, thereby making the filtering bleb dysfunctional [15, 16].

One solution to this problem is the combined procedure phacotrabeculectomy. Although in theory, it may possess many benefits, in reality, it obtained a poor reputation in its early years and is now a rarely used procedure in many glaucoma centers [17, 18]. However, the development of small incision phacoemulsification surgery has improved the success rates and reduced the complication rates after cataract surgery. This leads to the question—does modern cataract surgery allow a less hazardous profile of phacotrabeculectomy? The answer remains unclear and there is a lack of consensus on the best surgical management for these patients [19–22].

Here, we systematically reviewed the literature to compare the efficacy of phacotrabeculectomy with trabeculectomy (with or without later phacoemulsification surgery) on the management of glaucoma and coexisting cataract. We focused on small incision phacoemulsification surgery to present relevance to current clinical practice.

## 2. Materials and Methods

### 2.1. Study Design

This systematic review and meta-analysis was designed following the principles of the Grading of Recommendation, Assessment, Development, and Evaluation (GRADE) working group [23]. The topic was defined using the PICO approach which in short stands for the patient (P), intervention (I), comparison (C), and outcome (O) [24]. According to Danish law, no ethical committee or institutional review board approval was required for this study. We followed the items of the Preferred Reporting Items for Systematic Reviews and Meta-analyses (PRISMA) for all aspects of the reporting [25].

### 2.2. Eligibility Criteria and Outcome Measures

Eligible studies were defined as those who fulfilled the following criteria:

1. Population: patients with any type of glaucoma
2. Intervention: phacotrabeculectomy
3. Comparator: trabeculectomy with or without later phacoemulsification surgery
4. Outcomes: the primary outcome was the postoperative IOP closest to 12 months. Secondary outcomes were evaluated closest to 12 months and included visual acuity, visual field, the prevalence of complications with an exception for worsening of cataract, needling or revision, number of antiglaucomatous medications, surgical success, and failure
5. Study type: a comparative clinical study of humans. Studies were eligible regardless of study time (retrospective or prospective) or randomization

Intervention and/or comparator could be with or without the use of antimetabolites during surgery. We only considered studies disseminated in the English language. Unpublished registry trials were disregarded.

### 2.3. Information Sources, Search Strategy, and Study Selection

We searched the literature databases PubMed/MEDLINE, EMBASE, and the Cochrane Central. The search was performed on January 20, 2020. Considering the immense development in cataract surgery in the 20^th^ century and the differences between earlier practices and modern cataract surgery, we enforced a restriction on date of publication; i.e., we did not consider studies published prior to 1997 to ensure that only studies with modern surgical methods were included. Our search phrases and database searches were conducted with the assistance of a trained information specialist. We included a combination of keywords using the following search phrases:

1. (phaco-trabeculectomy OR phacotrabeculectomy) AND (“phacoemulsification”[Mesh] OR “Trabeculectomy”[Mesh] OR phacotrabeculectomy OR trabeculectomy OR phacoemulsification)
2. Trabeculectomy OR trabeculectomy failure OR trabeculectomy survival OR trabeculectomy success rate AND phacoemulsification AND (primary open-angle glaucoma OR POAG)

Two authors (A. A. and L. K.) screened titles and abstracts for eligibility and removed duplicates and obviously irrelevant reports. Remaining records were retrieved in full text to examine eligibility. All these records were read by two authors (A. A. and L. K.) who then discussed eligibility. In addition, reference lists of all articles read in the full text were crosschecked to identify other potentially relevant studies. Disagreements between the authors would lead to the involvement of a third author (D. B–H.) for further discussions and final decision making.

### 2.4. Data Collection and Risk of Bias Assessment

Two authors (A. A. and L. K.) extracted the following data from each eligible study: study design, study characteristics, glaucoma type, surgical methods, and outcomes of interest. The GRADEpro Guideline Development Tool was used to assess the quality of evidence for each outcome across studies [26]. The quality of evidence for each outcome started out as high level and could subsequently be downgraded because of limitations in study design (e.g., lack of randomization), risk of bias [27], inconsistency (heterogeneity) [28], indirectness [29], imprecision [30], and publication bias [31] to moderate, low, or even very low quality of evidence.

### 2.5. Data Synthesis and Analysis

All eligible studies were reviewed qualitatively in text and tables. The Review Manager 5.3 Software [32] was used to calculate estimates of overall treatment effects, and random-effect models were used to calculate pooled estimates of effects. Continuous outcome data were analyzed using the mean differences (MDs) approach with 95% confidence intervals (CIs), and dichotomous outcomes data were analyzed using risk ratios (RRs) with 95% CI.

## 3. Results

### 3.1. Study Selection

**Figure 1:**
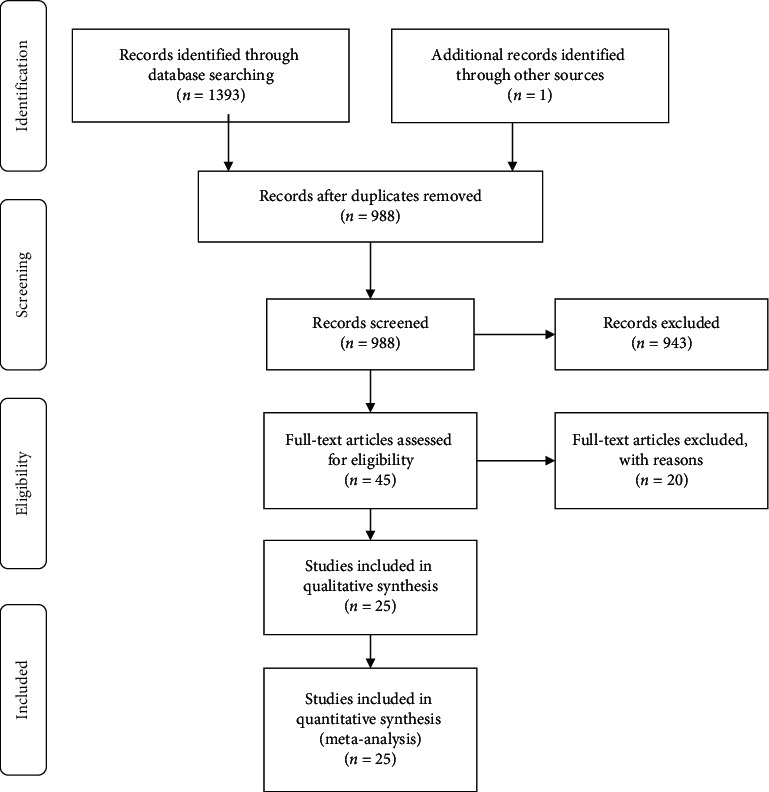
Flow diagram of the study selection process.

Our search strategy yielded a total of 1,393 records. We included one other study, which we knew of *a priori*. After removing the duplicates (*n* = 406), 988 records were screened using title and abstract, and 48 records were deemed to be of potential interest and retrieved in full text. Of these, 20 records were not eligible for our review (Supplementary file 1). We concluded that 25 studies were eligible for our qualitative and quantitative review (Figure 1).

### 3.2. Study and Population Characteristics

We identified studies comparing (a) phacotrabeculectomy (*n* = 2,315 eyes) with trabeculectomy (*n* = 2,216 eyes) and (b) phacotrabeculectomy (*n* = 75 eyes) with trabeculectomy followed by phacoemulsification performed 3–6 months after trabeculectomy (*n* = 71 eyes). We did not identify studies with other combinations of phacotrabeculectomy, trabeculectomy, and phacoemulsification.

We did not identify any randomized studies. We included 19 retrospective and six prospective studies. The majority of the studies included a mixed group of glaucoma subtypes, and six studies consisted of patients with POAG only. Studies were based on populations in North and South America (USA, *n* = 3; Canada, *n* = 2; Chile, *n* = 1), Australia, *n* = 1, Europe (UK, *n* = 2; Italy, *n* = 2; Switzerland, *n* = 1; Belgium, *n* = 1; Turkey, *n* = 1), and Asia (China, *n* = 3; Singapore, *n* = 1; Japan, *n* = 1; Hong-Kong, *n* = 1; South Korea, *n* = 2; Iran, *n* = 1; Israel, *n* = 1; Saudi Arabia, *n* = 1). A detailed description of the included studies is available in Table 1.

### 3.3. Primary Outcome: Postoperative IOP in Phacotrabeculectomy versus Trabeculectomy Only

**Figure 2:**
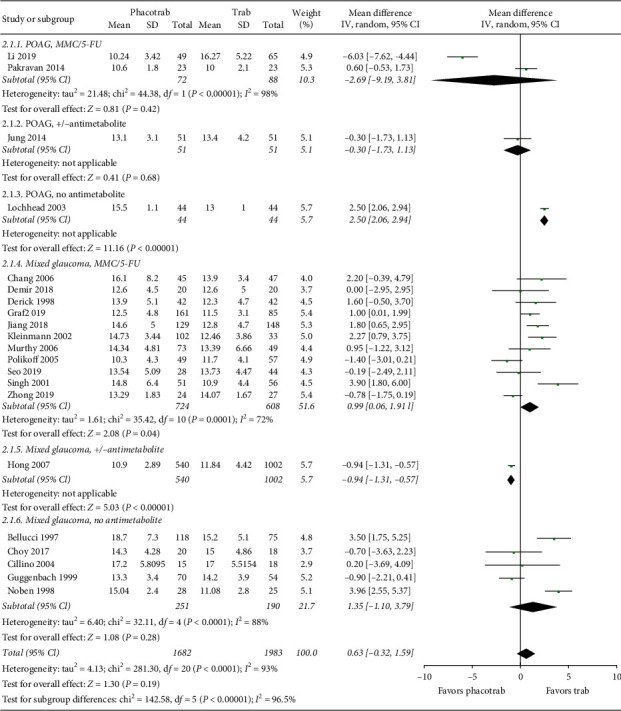
Forest plot of the IOP control at latest follow-up in eyes undergoing phacotrabeculectomy or trabeculectomy only. CI = confidence interval; d*f* = degrees of freedom; IV = inverse variance; SD = standard deviation. MMC = mitomycin c; 5-FU = 5-fluorouracil; +/− antimetabolite = not all eyes received antimetabolite during the procedure.

Twenty-one studies reported IOP control in patients undergoing phacotrabeculectomy versus trabeculectomy only. In total 1,682 eyes underwent phacotrabeculectomy versus 1,983 that received trabeculectomy. Evaluation of long-term IOP ranged from 1 month to 2 years in included studies [33–53] with 13 studies reporting IOP at 12 months after surgery [33, 36, 37, 39, 41, 43–45, 48, 49, 51–53]. Four studies included patients with POAG [33–36], and 17 studies included a mixed group of glaucoma patients [37–53]. The use of antimetabolites during the glaucoma procedures varied from the use of mitomycin C (MMC) or 5-fluorouracil (5-FU) or no use of antimetabolites to a combination of antimetabolites and no use of antimetabolites in the same study. Overall, we did not find any significant differences in long-term IOP control between the two groups, but the heterogeneity among studies was considerable (*I*^2^ = 93%) **(**Figure 2).

### 3.4. Primary Outcome: Postoperative IOP in Phacotrabeculectomy versus Phacoemulsification 3–6 Months after Trabeculectomy

**Figure 3:**
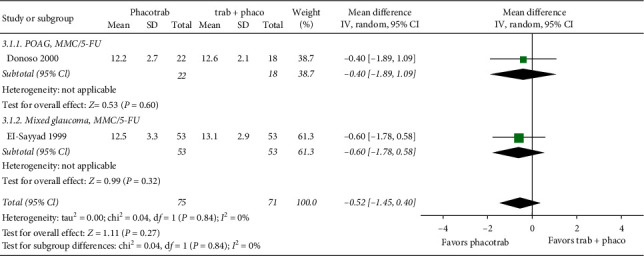
Forest plot of the IOP control postoperatively in eyes undergoing phacotrabeculectomy versus trabeculectomy with phacoemulsification 3–6 months later. CI = confidence interval; df = degrees of freedom; IV = inverse variance; SD = standard deviation. MMC = mitomycin c; 5-FU = 5-fluorouracil; ± antimetabolite = not all eyes received antimetabolite.

Two studies reported IOP in patients with POAG or mixed glaucoma undergoing phacotrabeculectomy (*n* = 75 eyes) or trabeculectomy followed by phacoemulsification (*n* = 71 eyes). All patients received perioperative antimetabolite (MMC or 5-FU). Postoperative IOP was measured at 12 months [57] or 2 years [56] after the last procedure. There was no difference in long-term IOP control between the two groups (Figure 3).

### 3.5. Secondary Outcome: Visual Acuity in Phacotrabeculectomy versus Trabeculectomy Only

**Figure 4:**
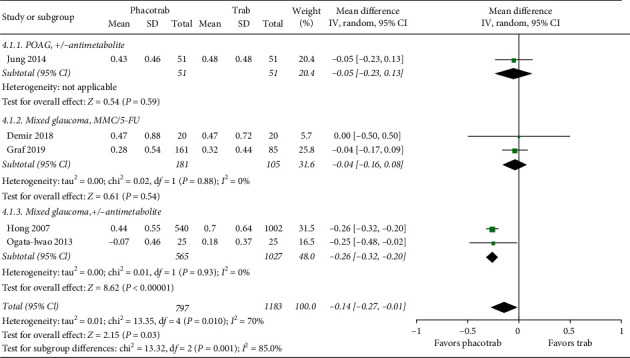
Forest plot of the visual acuity after phacotrabeculectomy versus trabeculectomy only. CI = confidence interval; d*f* = degrees of freedom; IV = inverse variance; SD = standard deviation. MMC = mitomycin c; 5-FU = 5-fluorouracil; +/− antimetabolite = not all eyes received antimetabolite.

Five studies reported logMAR visual acuity at any follow-up time in a manner that could be included in a meta-analysis. One study was based on patients with POAG [35], and the four other studies were based on a mixed glaucoma group [38, 40, 48, 55]. All studies used a combination of some patients receiving antimetabolites and others not receiving antimetabolites during glaucoma surgery. A total of 797 eyes had phacotrabeculectomy versus 1,183 who had trabeculectomy only. Long-term visual acuity was on average 0.14 logMAR better in the group receiving phacotrabeculectomy, corresponding to a 1.4-line difference on a visual acuity chart (*p*=0.03) (Figure 4).

### 3.6. Secondary Outcome: Prevalence of Complications in Phacotrabeculectomy versus Trabeculectomy Only

**Figure 5:**
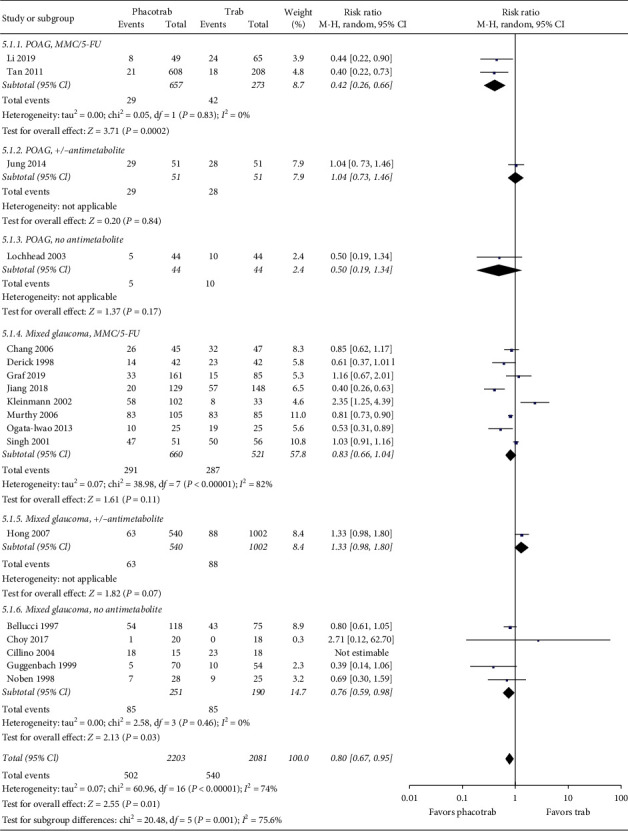
Forest plot of the risk of complications after phacotrabeculectomy versus trabeculectomy only. CI = confidence interval; d*f* = degrees of freedom; IV = inverse variance; SD = standard deviation. MMC = mitomycin c; 5-FU = 5-fluorouracil; ± antimetabolite = not all eyes received antimetabolite.

Eighteen studies reported complications at the latest reported follow-up in eyes undergoing phacotrabeculectomy or eyes receiving trabeculectomy only. Four studies were based on patients with POAG [33, 35, 36, 54]; the remaining 14 studies were based on a mixed glaucoma group [37, 39–43, 46, 48–53, 55]. The use of antimetabolites during the glaucoma procedures varied from the use of mitomycin C (MMC) or 5-fluorouracil (5-FU) or no use of antimetabolites to a combination of antimetabolites and no use of antimetabolites in the same study. The studies reported a wide range of complications ranging from less severe to very severe: hyphema, conjunctival scars, corneal edema, keratitis, postoperative IOP spike, bleb leak, flat/shallow anterior chamber, hypotony, hypotonous maculopathy, severe postoperative inflammation, fibrin reaction, iris prolapsed, lens malposition, blebitis, endophthalmitis, bleeding problems, posterior vitreous detachment, epiretinal membrane, retinal detachment, serous choroidal detachment, neovascular glaucoma, hemispheric vein occlusion to aqueous misdirection syndrome. The included studies reported a total of 502 complications in the 2,203 eyes undergoing phacotrabeculectomy (22.8%) versus 540 complications in the 2,081 eyes (25.9%) undergoing trabeculectomy only. The difference was statistically significant (RR = 0.80, 95% confidence interval: 0.67 to 0.95, *p*=0.01) (Figure 5).

### 3.7. Secondary Outcome: Prevalence of Complications in Phacotrabeculectomy versus Phacoemulsification 3–6 Months after Trabeculectomy

Prevalence of complications was evaluated in eyes undergoing phacotrabeculectomy (*n* = 28/75) or the consecutive procedure of trabeculectomy and phacoemulsification (*n* = 37/71) in patients with POAG or mixed glaucoma [56, 57]. All patients received perioperative antimetabolite (MMC or 5-FU). There was no significant difference in the risk of complications in eyes that had phacotrabeculectomy performed compared to the total number of complications in eyes that had a trabeculectomy followed by phacoemulsification (Supplementary Figure S1).

### 3.8. Secondary Outcome: Visual Field in Phacotrabeculectomy versus Trabeculectomy Only

Two studies [41, 48] reported the effects on visual fields in patients undergoing phacotrabeculectomy versus trabeculectomy only. The studies were based on a mixed group of glaucoma patients, some patients received antimetabolites, and some did not. In total, 669 eyes underwent phacotrabeculectomy versus 1,150 that received trabeculectomy. No significant difference was found between the two groups (Supplementary Figure S2).

### 3.9. Secondary Outcome: Needling or Revision in Phacotrabeculectomy versus Trabeculectomy Only

Nine studies reported the need for needling or revision. One study was based on patients with POAG [54], and the other studies were based on a mixed glaucoma group with a combination of some patients receiving antimetabolites and others not [37, 40, 41, 45, 46, 48, 50, 52]. 1,652 eyes received the combined procedure whereas 1,662 underwent trabeculectomy. No significant difference was found between the two groups (Supplementary Figure S3).

### 3.10. Secondary Outcome: Surgical Success in Phacotrabeculectomy versus Trabeculectomy Only

#### 3.10.1. Complete Success

Twelve studies reported complete success, which was obtained in a total of 951 out of 1,184 (80.3%) eyes undergoing phacotrabeculectomy and 1,375 out of 1,658 (82.9%) eyes undergoing trabeculectomy only. Two studies were based on patients with POAG [33, 35] and ten studies based on the mixed glaucoma group [37, 39–41, 44–46, 48, 50, 51]. The use of antimetabolites during surgery varied between the included studies. There was no significant difference between groups (Supplementary Figure S4). It should be noted that success criteria varied among included studies; a detailed description of success and failure criteria can be found in Table 1.

#### 3.10.2. Qualified Success

Qualified success was reported in 12 studies, and its definition varied among the studies (Table 1). A total of 708 out of 1,041 (68.0%) eyes undergoing phacotrabeculectomy had qualified surgical success versus 1,191 out of 1,597 (74.6%) of patients undergoing trabeculectomy only. Two studies were based on patients with POAG [33, 35] and ten studies based on the mixed glaucoma group [37, 39, 41, 44–46, 48, 50, 51, 55]. The use of antimetabolites during surgery varied between the included studies. There was no difference in the likelihood of qualified success between groups (Supplementary Figure S5).

### 3.11. Secondary Outcome: Surgical Failure in Phacotrabeculectomy versus Trabeculectomy Only

Surgical failure was reported in 11 studies [33, 35, 40, 41, 44–46, 48, 50, 53, 55]. Two of these studies were based on patients with POAG [33, 35]. The use of antimetabolites during surgery varied between the included studies. Failure was reported in 117 out of 1,121 (10.4%) eyes undergoing phacotrabeculectomy versus 130 out of 1,596 (8.1%) eyes undergoing trabeculectomy only. There was no significant difference between groups (Supplementary Figure S6).

### 3.12. Secondary Outcome: Number of Antiglaucomatous Medications in Phacotrabeculectomy versus Trabeculectomy Only

Eleven studies reported the number of antiglaucomatous medications in 1,130 eyes receiving phacotrabeculectomy and 1,438 receiving trabeculectomy only. The latest available follow-up from where data were extracted ranged from 3 months [40] to 2 years [48]. However, the majority of studies reported the status at 12 months after surgery [37, 39, 42, 46, 51–53, 55]. One study was based on POAG patients [35], while the remaining 10 studies were based on a mixed group of glaucoma patients; some patients received antimetabolites and others not [37, 39, 40, 42, 46, 48, 51–53, 55]. There was no difference in the number of antiglaucomatous medications when comparing data from those undergoing phacotrabeculectomy versus trabeculectomy only (Supplementary Figure S7).

### 3.13. Risk of Bias within Studies

The quality of evidence was rated as very low for all outcomes (Supplementary File S2). The quality of evidence was downgraded due to the lack of randomized trials. In addition, the 25 included trials differed considerably in study design as well as included patients (e.g., glaucoma subtypes), details regarding the procedure (e.g., use of antimetabolites), and definition of outcomes (e.g., the definition of surgical success). The majority of the included studies except two reported the postoperative IOP [33–53, 56]. Postoperative complications were reported by 20 studies [33, 35–37, 39–43, 46, 48–57]. Use of antiglaucomatous medication after surgery was reported by 12 studies [33, 35, 37, 39, 40, 42, 46, 48, 51–53, 55]. Several studies reported success criteria subdivided as complete [33, 35,37, 39–41, 44–46, 48, 50, 51], qualified [33, 35, 37, 39, 41, 44–46, 48, 50, 51, 55], and failure [33, 35, 40, 41, 44–46, 48, 50, 53, 55], but the definition of complete and qualified success and failure varied among studies; see Table 1. The need for needling or revision in the intervention groups was reported by nine studies [37, 40, 41, 45, 46, 48, 50, 52, 54]. Visual acuity was reported by five studies [35, 38, 40, 48, 55]. Furthermore, the quality of evidence was downgraded because only half of the outcomes met the optimum information size, which is the number of participants needed for analysis to show a difference at a certain power [30] which means that for the other half of the outcomes, too few patients had been included collectively by the studies analyzed to reach any certainty as to which intervention provided a better or worse outcome.

## 4. Discussion

In this systematic review with meta-analyses, we found no difference in postoperative IOP control between phacotrabeculectomy and trabeculectomy with or without later phacoemulsification, whereas the complication rate was significantly lower with phacotrabeculectomy. The IOP-lowering effect is important, as low IOP is the primary goal of glaucoma surgery. The surgical complication rate is obviously another crucial factor to consider when choosing which surgical method to use. Additionally, we found a positive effect on visual acuity after phacotrabeculectomy compared to trabeculectomy. This difference is not surprising, and a comparison of the change in visual acuity after a phacotrabeculectomy compared to trabeculectomy followed by phacoemulsification would be ideal, but unfortunately, these results were not available in the included studies. Other outcome measures (needling or revision, number of antiglaucomatous medications, and surgical success) 12 months postoperatively did not differ significantly between the groups. When interpreting these results, it is important to remember that this evidence is based on nonrandomized comparative studies with a marked risk of biases. However, we summarize the best evidence available, which suggests that phacotrabeculectomy for glaucoma in eyes with coexisting cataract should be considered a reasonable option. Well-designed randomized clinical trials are warranted for more conclusive evidence.

There were significantly fewer postoperative complications among those undergoing phacotrabeculectomy when compared to trabeculectomy with or without later phacoemulsification (22.8% versus 25.9%). Postoperative endophthalmitis was reported in seven studies [37, 41–43, 48, 53, 54] at a rate of 0.4% versus 0.3% in phacotrabeculectomy and trabeculectomy, respectively. One of the most frequently reported complications was hypotony. Twelve studies [35, 37, 39–41, 43, 46, 48, 50, 51, 54, 55] reported hypotony in a total of 123 out of 2,203 (5.6%) eyes undergoing phacotrabeculectomy and 184 out of 2,081 (8.8%) eyes undergoing trabeculectomy only. One could hypothesize that the greater inflammation after phacotrabeculectomy decreased the risk of hypotony.

Remarkably, only two studies reported the effect of surgery on visual field preservation, which makes it difficult to draw any credible conclusion on this important topic. This problem—a plethora of IOP data and absence of visual field data—is a well-known issue in many glaucoma studies and limits the generalizability of the conclusions of this study in terms of what to expect regarding postoperative preservation of visual field.

The likelihood of surgical success was only reported by studies comparing phacotrabeculectomy with trabeculectomy. There was no overall significant difference in the likelihood of surgical success between the two procedures. The criteria used to define complete and qualified success and failure varied considerably among the included studies making a comparison between studies challenging. However, the criteria for surgical success were the same for all participants in the individual studies, making the study-specific comparison usable. Differences in the definition of surgical success in glaucoma literature have been addressed previously. A systematic review with a search limit of 5 years found 92 IOP-related success definitions. When these criteria were applied to the same subset of eyes undergoing trabeculectomy, the success rate varied between 36 and 98% [58, 59].

Limitations of the present study should be taken into account when interpreting its results. First, our data is based on nonrandomized studies, which leads to a low evidence level for our conclusions. When patients are not randomized and data are obtained retrospectively, it should be remembered that the patient has been assigned to a certain intervention often based on what was considered to be the best option for the patient. This bias can only be addressed appropriately through prospectively designed randomized clinical trials. Second, the differences across studies in their design and definitions introduce a level of uncertainty when pooling data. This is unfortunately an issue in any systematic review, but within the field of glaucoma, there is an ambition of achieving stronger uniformity with the World Glaucoma Association Guidelines [60]. Hopefully, this limitation will be less of an issue in the future. Third, although we present analyses of different subtypes of glaucoma and use of metabolites separately, one limitation is that we look at different glaucoma subtypes collectively and not only on a specific subtype of glaucoma. This may introduce some uncertainty in the interpretation of the results. Fourth, although meta-analyses provide summary estimates of reported data and are high in the evidence pyramid, it should be remembered that the summary estimates in this study are a sum of nonrandomized comparative studies with important limitations. Therefore, our results should be interpreted with caution. Finally, to some extent, it is our perception that phacotrabeculectomy is a topic with different opinions. It can be speculated that such opinions influence publication decisions and therefore publication bias may be present.

## 5. Conclusions

We find similar postoperative IOP control, fewer complications, and better visual acuity with phacotrabeculectomy compared to trabeculectomy only. Phacotrabeculectomy addresses the patients´ two eye diseases simultaneously, possibly shortening the patients' contact to the health care system, and is a surgical option to consider when choosing the best surgical option for a patient with coexisting glaucoma and cataract and a need for an IOP-lowering procedure. Although this is the best evidence available, it should be noted that the level of evidence is low, based primarily on nonrandomized or retrospective studies, and better-designed studies are needed.

## Figures and Tables

**Table 1:** Description of included studies.

Study	Methods	Glaucoma	Exclusion criteria	Surgical method (*n*)	Outcome measures	Notes
Phacotrabeculectomy versus trabeculectomy						

Li et al. 2019 [33]	Retrospective review	POAG	Other eye diseases, serious cardiovascular, cerebrovascular diseases, diabetes, cancer, mental defects, physical disabilities; patients with surgical tolerance; patients who transferred to other hospitals during treatment	Phacotrabeculectomy (49) mean age 37.40 ± 10.90*y* Trabeculectomy (65) mean age 39.30 ± 11.90 *y* MMC	(1) IOP (12 months) (2) Visual acuity (12 months) (3) Complete success (IOP ≤ 21) (4) Qualified success (IOP > 21 mmHg, but decreased to ≤21 mmHg after taking IOP-lowering medication) (5) Failure (IOP > 21 mmHg	(2) Visual acuity not defined as LogMAR

Pakravan et al. 2014 [34]	Prospective comparative case series	POAG	History of contact lens wear, previous intraocular surgery, any corneal disease such as keratoconus, corneal dystrophies or corneal scars, CCT ≥ 580 or ≤ 500 microns, post-operative IOP ≥ 21 or ≤5 mmHg and occurrence of any surgical complications.	Phacotrabeculectomy (23) mean age 60.2 ± 20.2*y* Trabeculectomy (23) mean age 54.7 ± 24.2*y* MMC	(1) IOP (3 months)	—

Jung et al. 2014 [35]	Comparative retrospective consecutive case series review	POAG	Patients with glaucoma. Exclusion criteria prior filtering surgery, phacoemulsification in the previous 6 months, surgeries performed by residents in training.	Phacotrabeculectomy (51) mean age 73.7 ± 7.5 *y* Trabeculectomy (51) mean age 59.7 ± 9.8 *y* +/− anti-metabolite (1) Phacotrabeculectomy 0/51 received (2) Trabeculectomy 5/51 received	(1) IOP (6 months) (2) Log-MAR visual acuity (6 months) (3) Complications (4) Anti-glaucomatous medication (6 months) (5) Complete success (IOP < 21 mmHg /20% reduction on two consecutive follow-up visits after 3 months, without IOP-lowering medication) (6) Qualified success (IOP < 21 mmHg /20% reduction below baseline on two consecutive follow-up visits after 3 months with IOP-lowering medication) (7) Failure (IOP > 21 mmHg /<20% reduction below baseline on two consecutive follow-up visits after 3 months, IOP ≤ 5 mmHg on two consecutive visits after 3 months, reoperation for glaucoma, or loss of light perception vision)	—

Lochhead et al. 2003 [36]	Retrospective study	POAG	—	Phacotrabeculectomy (44) Trabeculectomy (44) No anti-metabolite No significant differences between groups with respect to age *p*=0.75	(1) IOP (12 months) (2) Complications (3) Anti-glaucomatous medication (4) Surgical success	(3) Anti-glaucomatous medication is only described as *p* value (0.4) (4) Graphs show results of surgical success but numbers could not be extracted

Chang et al. 2006 [37]	Retrospective, non-randomized study	POAG ACG CACG PXF PDG	Prior filtering surgery, excessive risk for conjunctival scarring	Phacotrabeculectomy (45) mean age 76.2 (58.0–94.0)*y* Trabeculectomy (47) mean age 70.0 (44.3–88.1)*y* 5-FU	(1) IOP (minimum 12 months) (2) Complications (3) Anti-glaucomatous medication (minimum 12 months) (4) Surgical success (5) Needling (6) Visual acuity	(6) Visual acuity means ± SDs could not be obtained from article.

Demir 2018 [38]	Prospective, non-randomized study	Mixed glaucoma	Optic disk anomaly except for glaucomatous changes, hereditary retinal diseases, retinal vascular diseases, fundus pathologies, those with low test reliability values, astigmatism higher than 3.00 D, cataract density higher than grade 2 according LOCS III, epinephrine corneal toxicity corneal disease and surgical history. Corneal endothelial density below 1300 cells/mm^2^ and those who failed to continue their postop checkups for at least 1 month.	Phacotrabeculectomy (20) mean age 66.2 ± 10.9*y* Trabeculectomy (20) mean age 61.6 ± 12.1*y* 5-FU	(1) IOP (1 month) (2) Log-MAR visual acuity (1 month)	—

Derick et al. 1998 [39]	Retrospective review	POAG PXF ACG Other	Observation <12 months	Phacotrabeculectomy (42) mean age 75.9 ± 8.0*y* Trabeculectomy (42) mean age 73.8 ± 9.3*y* MMC	(1) IOP (minimum 12 months) (2) Anti-glaucomatous medication (minimum 12 months) (3) Complete success (IOP < 21 mmHg, no IOP-lowering medication) (4) Qualified success (IOP < 22 mmHg, with IOP-lowering medication) (5) Complications (6) Visual acuity	(6) Visual acuity described as snellen acuity

Graf et al. 2019 [40]	Prospective study	POAG PEX NTG CAG SEC	—	Phacotrabeculectomy (161) mean age 74.7 ± 8.1*y* Trabeculectomy (85) mean age 69.0 ± 12.0*y* MMC	(1) IOP (24 months) (2) Log-MAR visual acuity (3 months) (3) Complete success (achieved target pressure according to visual field defects) (4) Failure (target pressure not achieved) (5) Anti-glaucomatous medication (3 months) (6) Complications (7) Needling	(1) No SD value for IOP at 3 months.

Jiang et al. 2018 [41]	Retrospective, cohort observational study	POAG PACG	Traumatic, neovascular, exfoliative, pigmentary, congenital and uveitic glaucoma	Phacotrabeculectomy (129) mean age 68.34 ± 11.4*y* Trabeculectomy (148) mean age 63.5 ± 11.1*y* MMC	(1) IOP (12 months) (2) Complete success (IOP < 20 mmHg and/or 20% reduction from baseline. (3) Qualified success (IOP < 20 mmHg, with single IOP-lowering medication) (4) Failure (requiring more than one topical agent and/or repeat surgery) (5) Complications (6) Needling (7) Visual acuity	(7) Visual acuity: SD value could not be obtained from article.

Kleinmann et al. 2002 [42]	Retrospective comparative study	POAG PXF ACG SEC	Surgery without use of MMC, prior ocular surgery	Phacotrabeculectomy (102) mean age 74.1 ± 6.8*y* Trabeculectomy (33) mean age 69.0 ± 14.6*y* MMC	(1) IOP (latest follow-up) (2) Anti-glaucomatous medication (latest follow-up) (3) Complications	—

Murthy et al. 2006 [43]	Retrospective, cohort study	POAG CACG PXF PDG NTG Neovascular uveitis-induced	Ocular conditions that could interfere with accurate assessment of IOP, including fuchs endothelial dystrophy and pseudophakic bullous keratopathy.	Phacotrabeculectomy (73) mean age 73.1 ± 12.4*y* Trabeculectomy (49) mean age 66.1 ± 16.7*y* MMC	(1) IOP (12 months) (2) Complications (3) Anti-glaucomatous medication (4) Surgical success	(3) Graphs show results of surgical success but numbers could not be extracted (4) Anti-glaucomatous medication no reported SD value

Polikoff et al. 2005 [44]	Retrospective review	POAG PXE Uveitis-induced LTG Congenital Neovascular PDG Traumatic	Eyes that required further surgical intervention within 1*y* from time of surgery, postoperative IOP > 21 mmHg.	Phacotrabeculectomy (53) mean age 70.3 ± 14.8*y* Trabeculectomy (82) mean age 60.5 ± 19.2*y* MMC/5-FU (1) IOP: Phacotrabeculectomy (49) Trabeculectomy (57) (2) Success: Phacotrabeculectomy (49) Trabeculectomy (72) (3) Failure: Phacotrabeculectomy (53) Trabeculectomy (82)	(1) IOP (12 months) (2) Complete success (IOP < 22 mmHg, no IOP-lowering medication, no further surgical interventions within 1 year) (3) Qualified success (IOP < 22 mmHg, with IOP-lowering medication, no further surgical interventions within 1 year) (4) Failure (IOP > 22 mmHg, further surgical interventions within 1 year	(1)–(4) All patients were not always included in the subsequent analyses.

Seo et al. 2019 [45]	Retrospective review	POAG PACG Secondary glaucoma	Patients unwilling to perform bleb OCT-A, missed followup visits, or had poor OCT-A image quality.	Phacotrabeculectomy (28) mean age 66.75 ± 7.77*y* Trabeculectomy (44) mean age 61.18 ± 12.97*y* MMC	(1) IOP (12 months (2) Complete success (IOP ≤ Target mmHg) (3) Qualified success (IOP ≤ 21 mmHg) (4) Failure (reoperation due to an increase in IOP despite medication and needling) (5) Needling	—

Singh et al. 2001 [46]	Retrospective, unmatched, non-randomized study	POAG PXF PDS CACG Traumatic Congenital Possner-Schlossman syndrome	—	Phacotrabeculectomy (51) mean age 76.2 ± 6.9*y* Trabeculectomy (56) mean age 63.3 ± 12.1*y* 5-FU	(1) IOP (24 months) (2) Complications (3) Anti-glaucomatous medication (24 months) (4) Complete success (IOP < 16 mmHg and a 30% reduction in IOP, no medication after 24 months) (5) qualified success (IOP < 16 mmHg and a 30% reduction in IOP, with medication after 24 months) (6) failure (IOP > 15 mmHg /< 30% reduction in IOP even with medications, or required revision surgery within 24 months) (7) Needling (8) Visual acuity	(8) Visual acuity described as snellen acuity change

Zhong et al. 2019 [47]	Retrospective, case-control study	POAG PACG	Ocular surface diseases, dry eyes according to the Japanese dry eye diagnostic criteria, ocular injury, infection, surgery and using contact lens, using drugs including preservative benzalkonium.	Phacotrabeculectomy (24) mean age 53.25 ± 3.40*y* Trabeculectomy (27) mean age 52.19 ± 3.28*y* 5-FU	(1) IOP (3 months)	—

Hong et al. 2007 [48]	Retrospective review	POAG CPACG	Less than 3*y* follow-up, other ocular diseases or intraocular surgical histories, other diseases affecting their visual fields or diabetes.	Phacotrabeculectomy (540) mean age 66.2 ± 11.0*y* Trabeculectomy (1002) mean age 51.4 ± 16.3*y* +/–MMC (1) Phacotrabeculectomy 88,77% reccevied (2) Trabeculectomy 91,11% reccevied	(3) IOP (12 months) (4) LogMAR visual acuity (at last success follow-up) (5) Complications (at last success follow-up) (6) Antiglaucomatous medication (at last success follow-up) (7) Failure (at last success follow-up) (8) Success ((a) IOP was reduced by at least 30% from baseline; (b) IOP ≤ 21 mmHg; (c) IOP < 18 mmHg) (9) Visual field (at last success follow-up) (10) Needling	(8) We considered success criteria c) as complete, criteria b) as qualified, and criteria a) as failure (∗) Postoperative values were at last successful follow-up (1) Phacotrabeculectomy 9.21 ± 4.86y (2) Trabeculectomy 10.74 ± 4.43y

Bellucci et al. 1997 [49]	Retrospective study	Mixed glaucoma	—	Phacotrabeculectomy (118) mean age 72 ± 10*y* Trabeculectomy (75) mean age 55 ± 16*y* no anti-metabolite	(1) IOP (12 months) (2) Complications (3) Anti-glaucomatous medication	(3) Anti-glaucomatous medication ± SDs could not be obtained from article.

Choy 2017 [50]	Retrospective review	POAG CACG Uveitic-glaucoma	—	Phacotrabeculectomy (20) mean age 65.7 ± 14.8*y* Trabeculectomy (18) mean age 62.4 ± 12.0*y* no anti-metabolite	(1) IOP (3 months) (2) Complications (3) Complete success (IOP < 22 mmHg and, no glaucoma medication) (4) Qualified success (IOP < 22 mmHg with the use of glaucoma medication) (5) Failure (IOP > 21 despite the use of glaucoma medications) (6) Needling (7) Visual acuity	(7) Visual acuity postoperatively described as acuity change

Cillino et al. 2004 [51]	Prospetive randomized clinical trial	POAG PEX	—	Phacotrabeculectomy (15) mean age 74.6 ± 1.1*y* Trabeculectomy (18) mean age 71.3 ± 1.2*y* no anti-metabolite	(1) IOP (12 months) (2) Complications (3) Complete success (IOP ≤ 21 mmHg and, no glaucoma medication) (4) Qualified success (IOP ≤ 21 mmHg with or without the use of glaucoma medication) (5) Anti-glaucomatous medication (12 months)	—

Guggenbach et al. 1999 [52]	Prospective study	POAG PXG	Previous surgery or laser trabeculoplasty performed 6 months or less before surgery, high risk patients primarily treated with antifibrotic agents at the time of surgery.	Phacotrabeculectomy (70) mean age 80.0 ± 7.0*y* Trabeculectomy (54) mean age 72.2 ± 9.0*y* no anti-metabolite	(1) IOP (12 months) (2) Complications (3) Anti-glaucomatous medication (12 months) (4) Needling (5) Visual acuity	(5) Visual acuity: Graphs show results but means ± SDs could not be obtained from article.

Noben et al. 1998 [53]	Retrospective review	POAG PXG PDS	Only one eye per patient was used. Use of anti-metabolite per- or postoperatively, previous glaucoma or other intraocular surgery.	Phacotrabeculectomy (28) mean age 76.17 (55–87)*y* Trabeculectomy (25) mean age 68.80 (52–83)*y* no anti-metabolite	(1) IOP (12 months) (2) Complications (3) Anti-glaucomatous medication (12 months) (4) Failure (IOP ≥ 21 mmHg or a less than 20% reduction from the preoperative level regardless the use of anti-glaucoma medications.	—

Tan et al. 2011 [54]	Retrospective review	POAG	Only one eye per patient was used. Surgery for acute primary angle closure.	Phacotrabeculectomy (608) mean age 70.3*y* Trabeculectomy (208) mean age 56.9 MMC/5-FU	(1) Complications (12 months) (2) Needling	—

Ogata-Iwao et al. 2013 [55]	Prospective study	POAG PXG	≤40*y*, IOP < 21 mmHg and history of ocular surgery.	Phacotrabeculectomy (25) mean age 73.1 ± 6.9*y* Trabeculectomy (25) mean age 72.1 ± 7.3*y* MMC	(1) Complications (2) Anti-glaucomatous medication (12 months) (3) Failure (IOP ≥ 26 mmHg at ≥ 3 months, despite completion of laser suture lysis and bleb needling) (4) Log-MAR visual acuity (12 months)	—
Post-operative IOP in patients undergoing phacotrabeculectomy versus phacoemulsification 3–6 months after trabeculectomy						

Donoso and Rodríguez2000 [56]	Retrospective review	POAG	Patients with glaucoma.	Phacotrabeculectomy (22) mean age 75.0 ± 5.0*y* Trabeculectomy + Phacoemulsification (18) mean age 78.0 ± 6.7*y* 5-FU	(1) IOP (phacotrabeculectomy 28.00 ± 16.14 months, trabeculectomy + phacoemulsification 21.35 ± 16.8 months) (2) Complications (3) Success (IOP < 20 mmHg at 12 months)	(3) Success, described as % and could not be converted to numerical proportions and not described by the other article.

El-Sayyad et al. 1999 [57]	Retrospective review	POAG CACG Combined-glaucoma PXG Steorid induced	Corneal opacities, subluxated cataractous lens, significant posterior segment disorders and/or previous eye surgery.	Phacotrabeculectomy (53) mean age 49.9 ± 14.3*y* Trabeculectomy + Phacoemulsification (53) mean age 50.3 ± 12.2*y* MMC/5-FU	(1) IOP (12 months) (2) Complications	(2) Complications are seen as total numbers according to the two groups.

POAG = primary open-angle glaucoma; PACG = primary angle-closure glaucoma; ACG = angle-closure glaucoma; CACG = chronic angle-closure glaucoma; CPACG = chronic primary angle-closure glaucoma; PGD = pigment dispersion glaucoma; PEX = pseudoexfoliation glaucoma; PXF = pseudoexfoliation glaucoma; NTG = normal-tension glaucoma; LTG = low tension glaucoma; SEC = secondary glaucoma.

## Data Availability

The original report data were obtained from the literature databases PubMed/MEDLINE, EMBASE, and the Cochrane Central.

## Supplementary Materials

Supplementary Materials Supplementary File 1: list of excluded studies. Supplementary File 2: a review of evidence quality. Supplementary Figure S1: forest plot of the risk of complications after phacotrabeculectomy versus phacoemulsification 3–6 months after trabeculectomy. Supplementary Figure S2: forest plot of the visual field after phacotrabeculectomy versus trabeculectomy only. Supplementary Figure S3: forest plot of the risk of needling or revision after phacotrabeculectomy versus trabeculectomy only. Supplementary Figure S4: forest plot of the complete success after phacotrabeculectomy versus trabeculectomy only. Supplementary Figure S5: forest plot of the qualified success after phacotrabeculectomy versus trabeculectomy only. Supplementary Figure S6: forest plot of the surgical failure after phacotrabeculectomy versus trabeculectomy only. Supplementary Figure S7: forest plot of the difference in a number of antiglaucomatous medications after phacotrabeculectomy versus trabeculectomy only.

## References

[1] Kingman S. (2004). Glaucoma is second leading cause of blindness globally. *Bulletin of the World Health Organization*.

[2] Foran S., Wang J. J., Mitchell P. (2003). Causes of visual impairment in two older population cross-sections: the blue mountains eye study. *Ophthalmic Epidemiology*.

[3] Wu S.-Y., Hennis A., Nemesure B., Leske M. C. (2008). Impact of glaucoma, lens opacities, and cataract surgery on visual functioning and related quality of life: the barbados eye studies. *Investigative Opthalmology & Visual Science*.

[4] Friedman D. S., Wolfs R. C., O’Colmain B. J. (2004). Prevalence of open-angle glaucoma among adults in the United States. *Archives of Ophthalmology (Chicago, Ill.: 1960)*.

[5] Congdon N (2004). Prevalence of cataract and pseudophakia/aphakia among adults in the United States. *Archives of Ophthalmology (Chicago, Ill.: 1960)*.

[6] Tham Y-C, Li X, Wong TY, Quigley HA, Aung T, Cheng C-Y (2014). Global prevalence of glaucoma and projections of glaucoma burden through 2040. *Ophthalmology*.

[7] Cordeiro M. F., Siriwardena D., Chang L., Khaw P. T. (2000). Wound healing modulation after glaucoma surgery. *Current Opinion in Ophthalmology*.

[8] Kung J. S., Choi D. Y., Cheema A. S., Singh K. (2015). Cataract surgery in the glaucoma patient. *Middle East African Journal of Ophthalmology*.

[9] Pohjalainen T., Vesti E., Uusitalo R. J., Laatikainen L. (2001). Phacoemulsification and intraocular lens implantation in eyes with open-angle glaucoma. *Acta Ophthalmologica Scandinavica*.

[10] Rajavi Z., Moezzi-Ghadim H., Kamrava K. (2009). The effect of trabeculectomy on cataract formation or progression. *Journal of Ophthalmic & Vision Research*.

[11] Daugeliene L., Yamamoto T., Sawada A., Kitazawa Y. (1998). An image analysis study of cataract development after trabeculectomy with mitomycin C. *Ophthalmologica*.

[12] Hylton C, Congdon N, Friedman D (2003). Cataract after glaucoma filtration surgery. *American Journal of Ophthalmology*.

[13] The AGIS Investigators (2001). The advanced glaucoma intervention study: 8. risk of cataract formation after trabeculectomy. *Archives of Ophthalmologyl*.

[14] Lichter P., Musch D. C., Gillespie B. W. (2001). Interim clinical outcomes in the collaborative initial glaucoma treatment study comparing initial treatment randomized to medications or surgery. *Ophthalmology*.

[15] Nishizawa A., Inoue T., Ohira S. (2016). The influence of phacoemulsification on surgical outcomes of trabeculectomy with mitomycin-C for uveitic glaucoma. *PLoS One*.

[16] Inoue T., Kawaji T., Inatani M., Kameda T., Yoshimura N., Tanihara H. (2012). Simultaneous increases in multiple proinflammatory cytokines in the aqueous humor in pseudophakic glaucomatous eyes. *Journal of Cataract & Refractive Surgery*.

[17] Augustinus C. J., Zeyen T. (2012). The effect of phacoemulsification and combined phaco/glaucoma procedures on the intraocular pressure in open-angle glaucoma. a review of the literature. *Bulletin de la Societe Belge d’ophtalmologie*.

[18] Tham C. C., Kwong Y. Y., Leung D. Y. (2010). Phacoemulsification vs phacotrabeculectomy in chronic angle-closure glaucoma with cataract: complications. *Archives of Ophthalmology*.

[19] Parihar J., Gupta R., Sahoo P. (2005). Phacotrabeculectomy versus conventional combined technique in coexisting glaucoma and cataract. *Medical Journal Armed Forces India*.

[20] Hsu C. H., Obstbaum S. A. (1998). Technique and outcome of combined phacoemulsification and trabeculectomy. *Current Opinion in Ophthalmology*.

[21] Friedman D. S., Jampel H. D., Lubomski L. H. (2002). Surgical strategies for coexisting glaucoma and cataract. *Ophthalmology*.

[22] Schuman J. S. (1996). Surgical management of coexisting cataract and glaucoma. *Ophthalmic Surgery and Lasers*.

[23] Guyatt G., Oxman A. D., Akl E. A. (2011). GRADE guidelines: 1. introduction-GRADE evidence profiles and summary of findings tables. *Journal of Clinical Epidemiology*.

[24] Guyatt G. H., Oxman A. D., Kunz R. (2011). GRADE guidelines: 2. framing the question and deciding on important outcomes. *Journal of Clinical Epidemiology*.

[25] Alderson D., Liberati A., Tetzlaff J., Altman D. G., PRISMA Group T. P. (2009). Preferred reporting items for systematic reviews and meta-analyses: the PRISMA statement. *PLoS Medicine*.

[26] Balshem H., Helfand M., Schünemann H. J. (2011). GRADE guidelines: 3. rating the quality of evidence. *Journal of Clinical Epidemiology*.

[27] Guyatt G. H., Oxman A. D., Vist G. (2011). GRADE guidelines: 4. rating the quality of evidence-study limitations (risk of bias). *Journal of Clinical Epidemiology*.

[28] Guyatt G. H., Oxman A. D., Kunz R. (2011). GRADE guidelines: 7. Rating the quality of evidence-inconsistency. *Journal of Clinical Epidemiology*.

[29] Guyatt G. H., Oxman A. D., Kunz R. (2011). GRADE guidelines: 8. Rating the quality of evidence-indirectness. *Journal of Clinical Epidemiology*.

[30] Guyatt G. H., Oxman A. D., Kunz R. (2011). GRADE guidelines 6. Rating the quality of evidence-imprecision. *Journal of Clinical Epidemiology*.

[31] Guyatt G. H., Oxman A. D., Montori V. (2011). GRADE guidelines: 5. Rating the quality of evidence-publication bias. *Journal of Clinical Epidemiology*.

[32] ReviewManager (RevMan). The Nordic Cochrane Centre Copenhagen, Denmark, 2014 TCC

[33] Li X., Liu Y., Li Y., Wang M. (2019). Effects of modified trabeculectomy combined with phacoemulsification and intraocular lens implantation on intraocular pressure and complications in patients with primary open angle glaucoma. *International Journal of Clinical and Experimental Medicine*.

[34] Pakravan M., Afroozifar M., Yazdani S. (2014). Corneal biomechanical changes following trabeculectomy, phaco-trabeculectomy, ahmed glaucoma valve implantation and phacoemulsification. *Journal of Ophthalmic & Vision Research*.

[35] Jung J. L., Isida-Llerandi C. G., Lazcano-Gomez G., SooHoo J. R., Kahook M. Y. (2014). Intraocular pressure control after trabeculectomy, phacotrabeculectomy and phacoemulsification in a hispanic population. *Journal of Current Glaucoma Practice*.

[36] Lochhead J., Casson R. J., Salmon J. F. (2003). Long term effect on intraocular pressure of phacotrabeculectomy compared to trabeculectomy. *British Journal of Ophthalmology*.

[37] Chang L., Thiagarajan M., Moseley M. (2006). Intraocular pressure outcome in primary 5FU phacotrabeculectomies compared with 5FU trabeculectomies. *Journal of Glaucoma*.

[38] Demir A. G., Olgun A., Guven D. (2018). The effect of combined phacotrabeculectomy, trabeculectomy and phacoemulsification on the corneal endothelium in the early stage: a preliminary study. *International Ophthalmology*.

[39] Derick R. J., Evans J., Baker N. D. (1998). Combined phacoemulsification and trabeculectomy versus trabeculectomy alone: a comparison study using mitomycin-C. *Ophthalmic Surgery and Lasers*.

[40] Graf N E., Müller M., Gerlach F. (2019). Comparison of 2-year-results of mitomycin C-augmented trabeculectomy with or without cataract extraction in glaucoma patients. *Canadian Journal of Ophthalmology*.

[41] Jiang L., Eaves S., Dhillon N., Ranjit P. (2018). Postoperative outcomes following trabeculectomy and nonpenetrating surgical procedures: a 5-year longitudinal study. *Clinical Ophthalmology*.

[42] Kleinmann G., Katz H., Pollack A., Schechtman E., Rachmiel R., Zalish M. (2002). Comparison of trabeculectomy with mitomycin C with or without phacoemulsification and lens implantation. *Ophthalmic Surgery Lasers*.

[43] Murthy S. K., Damji K. F., Pan Y., Hodge W. G. (2006). Trabeculectomy and phacotrabeculectomy, with mitomycin-C, show similar two-year target IOP outcomes. *Canadian Journal of Ophthalmology*.

[44] Polikoff L. A., Taglienti A., Chanis R. A. (2005). Is intraocular pressure in the early postoperative period predictive of antimetabolite-augmented filtration surgery success?. *Journal of Glaucoma*.

[45] Seo J. H., Lee Y., Shin J. H., Kim Y. A., Park K. H. (2019). Comparison of conjunctival vascularity changes using optical coherence tomography angiography after trabeculectomy and phacotrabeculectomy. *Graefe’s Archive for Clinical and Experimental Ophthalmology*.

[46] Singh R. P., Goldberg I., Mohsin M. (2001). The efficacy and safety of intraoperative and/or postoperative 5-fluorouracil in trabeculectomy and phacotrabeculectomy. *Clinical and Experimental Ophthalmology*.

[47] Zhong S., Zhou H., Chen X., Zhang W., Yi L. (2019). Influence of glaucoma surgery on the ocular surface using oculus keratograph. *International Ophthalmology*.

[48] Hong S., Park K., Ha S. J., Yeom H. Y., Seong G. J., Hong Y. J. (2007). Long-term intraocular pressure control of trabeculectomy and triple procedure in primary open angle glaucoma and chronic primary angle closure glaucoma. *Ophthalmologica*.

[49] Bellucci R., Perfetti S., Babighian S., Morselli S., Bonomi L. (1997). Filtration and complications after trabeculectomy and after phaco-trabeculectomy. *Acta Ophthalmologica Scandinavica. Supplement*.

[50] Choy B. N. K. (2017). Comparison of surgical outcome of trabeculectomy and phacotrabeculectomy in Chinese glaucoma patients. *International Journal of Ophthalmology*.

[51] Cillino S., Pace F. D., Casuccio A. (2004). Deep sclerectomy versus punch trabeculectomy with or without phacoemulsification. *Journal of Glaucoma*.

[52] Guggenbach M, Mojon DS, Bohnke M (1999). Evaluation of phacotrabeculectomy versus trabeculectomy alone. *Ophthalmologica*.

[53] Noben K. J., Linsen M. C., Zeyen T. G. (1998). Is combined phacoemulsification and trabeculectomy as effective as trabeculectomy alone?. *Bulletin de la Societe Belge d’ophtalmologie.*.

[54] Tan Y.-L., Tsou P. F., Tan G. S. (2011). Postoperative complications after glaucoma surgery for primary angle-closure glaucoma vs primary open-angle glaucoma. *Archives of Ophthalmology*.

[55] Ogata-Iwao M., Inatani M., Takihara Y., Inoue T., Iwao K., Tanihara H. (2013). A prospective comparison between trabeculectomy with mitomycin C and phacotrabeculectomy with mitomycin C. *Acta Ophthalmologica*.

[56] Donoso R., Rodríguez A. (2000). Combined versus sequential phacotrabeculectomy with intraoperative 5-fluorouracil. *Journal of Cataract & Refractive Surgery*.

[57] El-Sayyad F. F., Helal M. H., Khalil M. M., El-Maghraby M. A. (1999). Phacotrabeculectomy versus two-stage operation: a matched study. *Ophthalmic Surgery Lasers*.

[58] Morgan W. H., Yu D.-Y. (2012). Surgical management of glaucoma: a review. *Clinical & Experimental Ophthalmology*.

[59] Rotchford A. P., King A. J. (2010). Moving the goal posts. *Ophthalmology*.

[60] Heuer D. K., Grehn K. B. (2009). *Consensus on Definitions of Success Guidelines on Design and Reporting of Glaucoma Surgical Trials*.

