# Supplementary material for: Comparative Efficacy of Phacotrabeculectomy versus Trabeculectomy with or without Later Phacoemulsification: A Systematic Review with Meta-Analyses
Source: J Ophthalmol. 2021 Feb 13;2021:6682534. doi: 10.1155/2021/6682534 (PMC7896844; doi:10.1155/2021/6682534)
Supplement: Supplementary Materials — Supplementary File 1: list of excluded studies. Supplementary File 2: a review of evidence quality. Supplementary Figure S1: forest plot of the risk of complications after phacotrabeculectomy versus phacoemulsification 3–6 months after trabeculectomy. Supplementary Figure S2: forest plot of the visual field after phacotrabeculectomy versus trabeculectomy only. Supplementary Figure S3: forest plot of the risk of needling or revision after phacotrabeculectomy versus trabeculectomy only. Supplementary Figure S4: forest plot of the complete success after phacotrabeculectomy versus trabeculectomy only. Supplementary Figure S5: forest plot of the qualified success after phacotrabeculectomy versus trabeculectomy only. Supplementary Figure S6: forest plot of the surgical failure after phacotrabeculectomy versus trabeculectomy only. Supplementary Figure S7: forest plot of the difference in a number of antiglaucomatous medications after phacotrabeculectomy versus trabeculectomy only. [file 6682534.f1.zip › Supplementary file 1_list of excluded studies_new references.docx]

**Supplementary file 1:** List of excluded studies

| **Study** | **Reason for Exclusion** |
| --- | --- |
| Inatani 2000[1] | Compared trabeculotomy with phacotrabeculectomy. |
| Tanito April 2001[2] | Compared combined trabeculotomy and cataract surgery with cataract surgery. |
| Tanito Nov 2001[3] | Compared combined trabeculotomy and cataract surgery with cataract surgery. |
| Takihara 2011[4] | Compared trabeculectomy in phakic vs pseudophakic eyes. |
| Takihara 2014 [5] | Compared trabeculectomy in phakic vs pseudophakic eyes. |
| Popa-Cherecheanu 2017[6] | Compared phacoemulsification before trabeculectomy vs trabeculectomy after phacoemulsification. |
| Li 2016[7] | Compared phacotrabeculectomy with phacoemulsification. |
| Dhalla 2017[8] | Compared phacotrabeculectomy with phacoemulsification. |
| Bayer 2009[9] | Compared phacotrabeculectomy with microincision cataract surgery and trabeculectomy. |
| Parihar 2005[10] | Compared phacotrabeculectomy with combined extracapsular lens extraction with trabeculectomy. |
| Kaplan-Messas 2009[11] | Compared trabeculectomy and phacotrabeculectomy with and without peripheral iridectomy. |
| Sharma 2007[12] | Compared the role of 5-fluorouracil in patients receiving phacoemulsification after previous trabeculectomy. |
| Bowman 2010[13] | Comparison of phacotrabeculectomy with small incision cataract surgery trabeculectomy (SICST) and extra-capsular cataract extraction trabeculectomy (ECCET) |
| Shahid 2010[14] | Compared the role of 5-fluorouracil injection in patients receiving phacoemulsification after trabeculectomy. |
| Shin 2002[15] | Compared the role of Mitomycin-C in patients receiving phacotrabeculectomy. |
| Shin 1998[16] | Compared the role of Mitomycin-C in patients receiving phacotrabeculectomy. |
| Nguyen 2014 [17] | Compared patients who had phacoemulsification subsequent to trabeculectomy and patients who were pseudophakic preceding their trabeculectomy. |
| Awai-Kasaoka 2012[18] | Evaluated the impact of phacoemulsification after trabeculectomy |
| Shin 1998[19] | Comparisons between secondary glaucoma triple procedure (SGTP) and primary glaucoma triple procedure (PGTP) |
| Ren 1998[20] | Compared outcome of primary glaucoma triple procedure (PGTP) with adjunctive 5-fluorouracil. |

**References**

1. Inatani M, Tanihara H, Muto T, Honjo M, Okazaki K, Kido N, et al. Transient intraocular pressure elevation after trabeculotomy and its occurrence with phacoemulsification and intraocular lens implantation. Jpn J Ophthalmol. 2001/05/23. 2001;45:288–92.

2. Tanito M, Ohira A, Chihara E. Factors leading to reduced intraocular pressure after combined trabeculotomy and cataract surgery. J Glaucoma. 2002/02/01. 2002;11:3–9.

3. Tanito M, Ohira A, Chihara E. Surgical outcome of combined trabeculotomy and cataract surgery. J Glaucoma. 2001/09/18. 2001;10:302–8.

4. Takihara Y, Inatani M, Seto T, Iwao K, Iwao M, Inoue T, et al. Trabeculectomy with mitomycin for open-angle glaucoma in phakic vs pseudophakic eyes after phacoemulsification. Arch Ophthalmol. 2011/02/16. 2011;129:152–7.

5. Takihara Y, Inatani M, Ogata-Iwao M, Kawai M, Inoue T, Iwao K, et al. Trabeculectomy for open-angle glaucoma in phakic eyes vs in pseudophakic eyes after phacoemulsification: a prospective clinical cohort study. JAMA Ophthalmol. 2013/11/16. 2014;132:69–76.

6. Popa-Cherecheanu A, Iancu RC, Schmetterer L, Pirvulescu R, Coviltir V. Intraocular Pressure, Axial Length, and Refractive Changes after Phacoemulsification and Trabeculectomy for Open-Angle Glaucoma. J Ophthalmol. 2017/07/01. 2017;2017:1203269.

7. Li HJ, Xuan J, Zhu XM, Xie L. Comparison of phacotrabeculectomy and sequential surgery in the treatment of chronic angle-closure glaucoma coexisted with cataract. Int J Ophthalmol. 2016/06/09. 2016;9:687–92.

8. Dhalla K, Cousens S, Murdoch IE. Phacoemulsification compared with phacotrabeculectomy surgery: a within-person observational cohort study. Br J Ophthalmol. 2017/04/19. 2017;

9. Bayer A, Erdem U, Mumcuoglu T, Akyol M. Two-site phacotrabeculectomy versus bimanual microincision cataract surgery combined with trabeculectomy. Eur J Ophthalmol. 2009/01/06. 2009;19:46–54.

10. Parihar JKS, Gupta RP, Sahoo PK, Misra RP, Vats DP, Kamath AP, et al. Phacotrabeculectomy Versus Conventional Combined Technique in Coexisting Glaucoma and Cataract. Med J Armed Forces India [Internet]. 2005/02/01. 2005;61:139–42.

11. Kaplan-Messas A, Cohen Y, Blumenthal EZ, Avni I. Trabeculectomy and photo-trabeculectomy with and without peripheral iridectomy. Eur J Ophthalmol [Internet]. 2009;19:231–4.

12. Sharma TK, Arora S, Corridan PG. Phacoemulsification in patients with previous trabeculectomy: role of 5-fluorouracil. Eye. 2006/03/18. 2007;21:780–3.

13. Bowman RJC, Hay A, Wood ML, Murdoch IE. Combined cataract and trabeculectomy surgery for advanced glaucoma in East Africa; visual and intra-ocular pressure outcomes. Eye [Internet]. 2009/06/13. 2010;24:573–7.

14. Shahid H, Salmon JF. Use of 5-Fluorouracil injections to reduce the risk of trabeculectomy bleb failure after cataract surgery. J Ocul Pharmacol Ther. 2010/02/13. 2010;26:119–23.

15. Shin DH, Iskander NG, Ahee JA, Singal IP, Kim C, Hughes BA, et al. Long-term filtration and visual field outcomes after primary glaucoma triple procedure with and without mitomycin-C. Ophthalmology. 2002/09/05. 2002;109:1607–11.

16. Shin DH, Ren J, Juzych MS, Hughes BA, Kim C, Song MS, et al. Primary glaucoma triple procedure in patients with primary open-angle glaucoma: the effect of mitomycin C in patients with and without prognostic factors for filtration failure. Am J Ophthalmol. 1998/03/25. 1998;125:346–52.

17. Nguyen DQ, Niyadurupola N, Tapp RJ, O’Connell RA, Coote MA, Crowston JG. Effect of phacoemulsification on trabeculectomy function. Clin Exp Ophthalmol. 2013/12/19. 2014;42:433–9.

18. Awai-Kasaoka N, Inoue T, Takihara Y, Kawaguchi A, Inatani M, Ogata-Iwao M, et al. Impact of phacoemulsification on failure of trabeculectomy with mitomycin-C. J Cataract Refract Surg. 2011/12/23. 2012;38:419–24.

19. Shin DH, Kim YY, Sheth N, Ren J, Shah M, Kim C, et al. The role of adjunctive mitomycin C in secondary glaucoma triple procedure as compared to primary glaucoma triple procedure. Ophthalmology. 1998/04/17. 1998;105:740–5.

20. Ren J, Shin DH, O’Grady JM, Kim YY, Juzych MS, Hughes BA, et al. Long-term outcome of primary glaucoma triple procedure with adjunctive 5-fluorouracil. Graefes Arch Clin Exp Ophthalmol. 1998/07/22. 1998;236:501–6.
