# Supplementary material for: Comparative Efficacy of Phacotrabeculectomy versus Trabeculectomy with or without Later Phacoemulsification: A Systematic Review with Meta-Analyses
Source: J Ophthalmol. 2021 Feb 13;2021:6682534. doi: 10.1155/2021/6682534 (PMC7896844; doi:10.1155/2021/6682534)
Supplement: Supplementary Materials — Supplementary File 1: list of excluded studies. Supplementary File 2: a review of evidence quality. Supplementary Figure S1: forest plot of the risk of complications after phacotrabeculectomy versus phacoemulsification 3–6 months after trabeculectomy. Supplementary Figure S2: forest plot of the visual field after phacotrabeculectomy versus trabeculectomy only. Supplementary Figure S3: forest plot of the risk of needling or revision after phacotrabeculectomy versus trabeculectomy only. Supplementary Figure S4: forest plot of the complete success after phacotrabeculectomy versus trabeculectomy only. Supplementary Figure S5: forest plot of the qualified success after phacotrabeculectomy versus trabeculectomy only. Supplementary Figure S6: forest plot of the surgical failure after phacotrabeculectomy versus trabeculectomy only. Supplementary Figure S7: forest plot of the difference in a number of antiglaucomatous medications after phacotrabeculectomy versus trabeculectomy only. [file 6682534.f1.zip › Supplementary file 2. Review of evidence quality..docx]

**Supplementary file 2.** Review of evidence quality.

| **Certainty assessment** | | | | | | | **№ of patients** | | **Effect** | | **Certainty** |
| --- | --- | --- | --- | --- | --- | --- | --- | --- | --- | --- | --- |
| **№ of studies** | **Study design** | **Risk of bias** | **Inconsistency** | **Indirectness** | **Imprecision** | **Other considerations** | **Phaco-trab** | **Trab** | **Relative (95% CI)** | **Absolute (95% CI)** |  |
| **Phacotrabeculectomy versus trabeculectomy** | | | | | | | | | | | |
| IOP at latest follow-up | | | | | | | | | | | |
| 21 | Observational studies | serious ^a^ | very serious ^b^ | not serious | not serious ^c^ | publication bias strongly suspected ^d^ | 1682 | 1983 | - | MD **0.6**3 **higher** (0.32 lower to 1.59 higher) | ⨁◯◯◯ VERY LOW |
| logMAR visual acuity, latest follow-up | | | | | | | | | | | |
| 5 | Observational studies | serious ^a^ | serious ^e^ | not serious | not serious ^c^ | publication bias strongly suspected strong association ^f^ | 797 | 1183 | - | MD **0.14 lower** (0.27 lower to 0.01 lower) | ⨁◯◯◯ VERY LOW |
| Any complications, latest follow-up times | | | | | | | | | | | |
| 18 | Observational studies | serious ^a^ | serious ^g^ | not serious | serious ^h^ | publication bias strongly suspected ^i^ | 494/2203 (22.4%) | 534/2081 (25.7%) | RR 0.80 (0.67 to 0.95) | **51 fewer per 1.000** (from 85 fewer to 13 fewer) | ⨁◯◯◯ VERY LOW |
| Number of anti-glaucoma medications, latest follow-up | | | | | | | | | | | |
| 11 | Observational studies | serious ^a^ | serious ^j^ | not serious | not serious ^c^ | publication bias strongly suspected ^k^ | 1130 | 1438 | - | MD **0.05 higher** (0.06 lower to 0.15 higher) | ⨁◯◯◯ VERY LOW |
| Complete success | | | | | | | | | | | |
| 12 | Observational studies | serious ^a^ | serious^l^ | not serious | serious ^h^ | publication bias strongly suspected ^m^ | 951/1184 (80.3%) | 1375/1658 (82.9%) | RR 1.01 (0.93 to 1.09) | **8 more per 1.000** (from 58 fewer to 75 more) | ⨁◯◯◯ VERY LOW |
| Qualified success | | | | | | | | | | | |
| 12 | Observational studies | serious ^a^ | serious ^n^ | not serious | not serious ^c^ | publication bias strongly suspected ^o^ | 708/1041 (68.0%) | 1191/1597 (74.6%) | RR 1.00 (0.95 to 1.06) | **0 fewer per 1.000** (from 37 fewer to 45 more) | ⨁◯◯◯ VERY LOW |
| Failure | | | | | | | | | | | |
| 11 | Observational studies | serious ^a^ | serious ^p^ | not serious | serious ^h^ | publication bias strongly suspected ^q^ | 117/1121(10.4%) | 130/1596 (8.1%) | RR 0,98 (0.68 to 1.42) | **2 fewer per 1.000** (from 26 fewer to 34 more) | ⨁◯◯◯ VERY LOW |
| Visual fields, latest follow-up | | | | | | | | | | | |
| 2 | Observational studies | serious ^a^ | very serious ^r^ | not serious | not serious ^c^ | publication bias strongly suspected ^s^ | 669 | 1150 | - | MD **2.71 higher** (0.74 lower to 6.15 higher) | ⨁◯◯◯ VERY LOW |
| Needling or revision, latest follow-up | | | | | | | | | | | |
| 9 | Observational studies | serious ^a^ | not serious^t^ | not serious | not serious ^c^ | publication bias strongly suspected ^u^ | 128/1652 (7.7%) | 201/1662 (12.1%) | RR 1.03 (0.72 to 1.45) | **4 more per 1.000** (from 34 fewer to 54 more) | ⨁◯◯◯ VERY LOW |

**CI:** Confidence interval; **MD:** Mean difference; **RR:** Risk ratio

#### Explanations

a) Most information are from studies at moderate risk of bias. b) I^2^ = 93% c) Optimal information size was met.
d) Postoperative IOP were not reported by 2 studies e) I^2^ = 70%
f) Only 5 out of 23 studies reported visual acuity in logMAR values that could be used in the statistical calculations.
g) I^2^ = 74% h) Optimal information size was not met.
i) Complications were not reported by 4 studies. j) I^2^ = 58%
k) Use of postoperative medication were not reported by 12 studies. l) I^2^ = 54%
m) Complete success was only reported by 12 studies and each study used their own definition of success.
n) I^2^ = 30% o) Qualified success was only reported by 12 studies and each study used their own definition of success.
p) I^2^ = 43% q) Failure was only reported by 11 studies and each study used their own definition of failure.
r) I^2^ = 91.1% s) Visual fields was only reported by 2 studies out of 23
t) I^2^ = 0% u) Only 9 out of 23 studies reported on the necessity of needling or revision.

| **Certainty assessment** | | | | | | | **№ of patients** | | **Effect** | | **Certainty** |
| --- | --- | --- | --- | --- | --- | --- | --- | --- | --- | --- | --- |
| **№ of studies** | **Study design** | **Risk of bias** | **Inconsistency** | **Indirectness** | **Imprecision** | **Other considerations** | **Phaco-trab** | **Phacoemulsification 3-6 months after trab** | **Relative (95% CI)** | **Absolute (95% CI)** |  |
| **Phacotrabeculectomy compared to phacoemulsification 3-6 months after trabeculectomy** | | | | | | | | | | | |
| IOP at latest follow-up | | | | | | | | | | | |
| 2 | Observational studies | serious a | not serious | not serious | serious b | none | 75 | 71 | - | MD **0.52 lower** (1.45 lower to 0.4 higher) | ⨁◯◯◯ VERY LOW |
| Any complications, latest follow-up | | | | | | | | | | | |
| 2 | Observational studies | serious a | not serious | not serious | serious b | strong association | 28/75 (37.3%) | 37/71 (52.1%) | RR 0.76 (0.56 to1.03) | **125 fewer per 1.000** (from 229 fewer to 16 more) | ⨁◯◯◯ VERY LOW |

**CI**: Confidence interval; **RR:** Risk ratio

#### Explanations

a) Most information are from studies at moderate risk of bias. b) Optimal information size was not met.
