# Supplementary figures and images for: Comparative Efficacy of Phacotrabeculectomy versus Trabeculectomy with or without Later Phacoemulsification: A Systematic Review with Meta-Analyses
Source: J Ophthalmol. 2021 Feb 13;2021:6682534. doi: 10.1155/2021/6682534 (PMC7896844; doi:10.1155/2021/6682534)

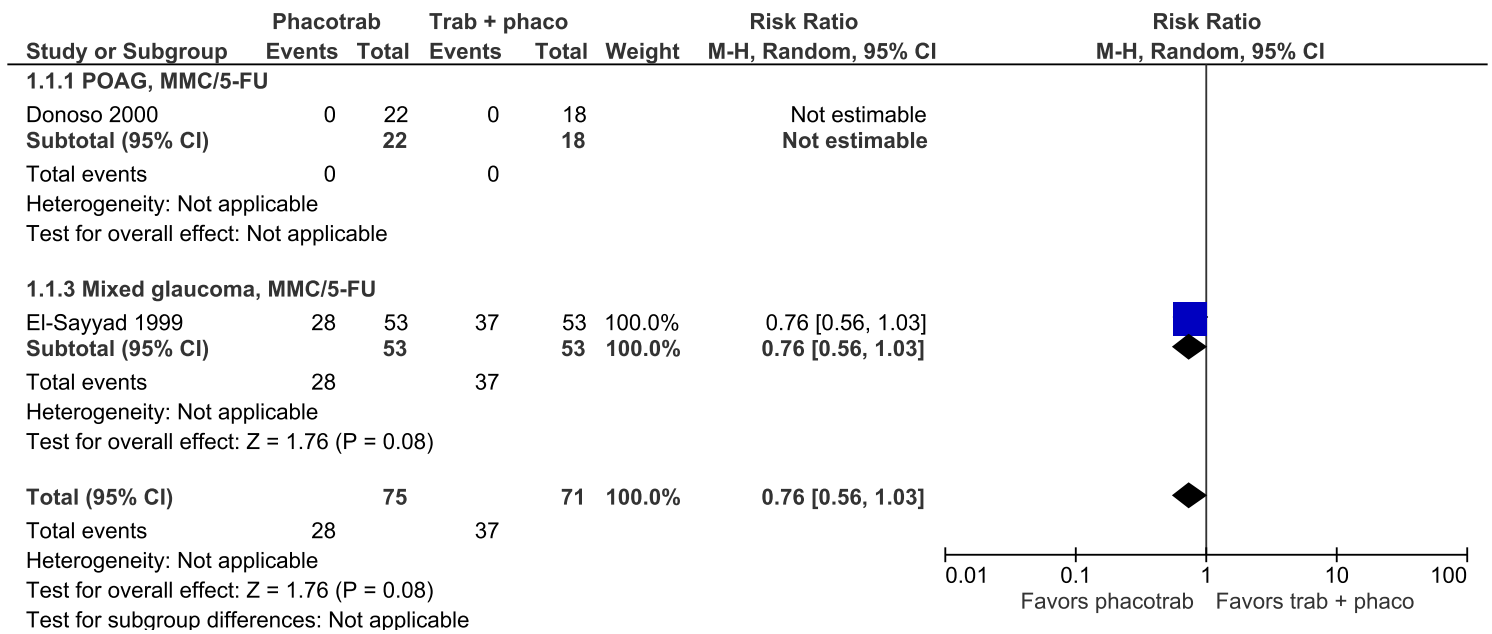

Supplement: Supplementary Materials — Supplementary File 1: list of excluded studies. Supplementary File 2: a review of evidence quality. Supplementary Figure S1: forest plot of the risk of complications after phacotrabeculectomy versus phacoemulsification 3–6 months after trabeculectomy. Supplementary Figure S2: forest plot of the visual field after phacotrabeculectomy versus trabeculectomy only. Supplementary Figure S3: forest plot of the risk of needling or revision after phacotrabeculectomy versus trabeculectomy only. Supplementary Figure S4: forest plot of the complete success after phacotrabeculectomy versus trabeculectomy only. Supplementary Figure S5: forest plot of the qualified success after phacotrabeculectomy versus trabeculectomy only. Supplementary Figure S6: forest plot of the surgical failure after phacotrabeculectomy versus trabeculectomy only. Supplementary Figure S7: forest plot of the difference in a number of antiglaucomatous medications after phacotrabeculectomy versus trabeculectomy only. [file 6682534.f1.zip › S1.pdf]

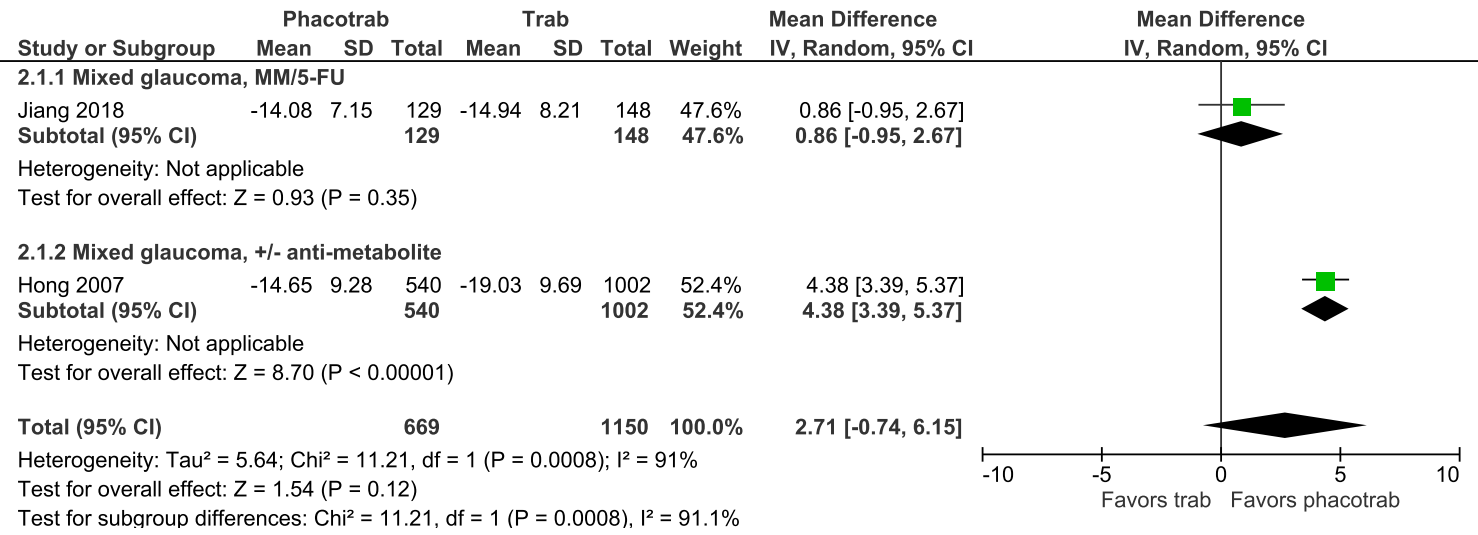

Supplement: Supplementary Materials — Supplementary File 1: list of excluded studies. Supplementary File 2: a review of evidence quality. Supplementary Figure S1: forest plot of the risk of complications after phacotrabeculectomy versus phacoemulsification 3–6 months after trabeculectomy. Supplementary Figure S2: forest plot of the visual field after phacotrabeculectomy versus trabeculectomy only. Supplementary Figure S3: forest plot of the risk of needling or revision after phacotrabeculectomy versus trabeculectomy only. Supplementary Figure S4: forest plot of the complete success after phacotrabeculectomy versus trabeculectomy only. Supplementary Figure S5: forest plot of the qualified success after phacotrabeculectomy versus trabeculectomy only. Supplementary Figure S6: forest plot of the surgical failure after phacotrabeculectomy versus trabeculectomy only. Supplementary Figure S7: forest plot of the difference in a number of antiglaucomatous medications after phacotrabeculectomy versus trabeculectomy only. [file 6682534.f1.zip › S2.pdf]

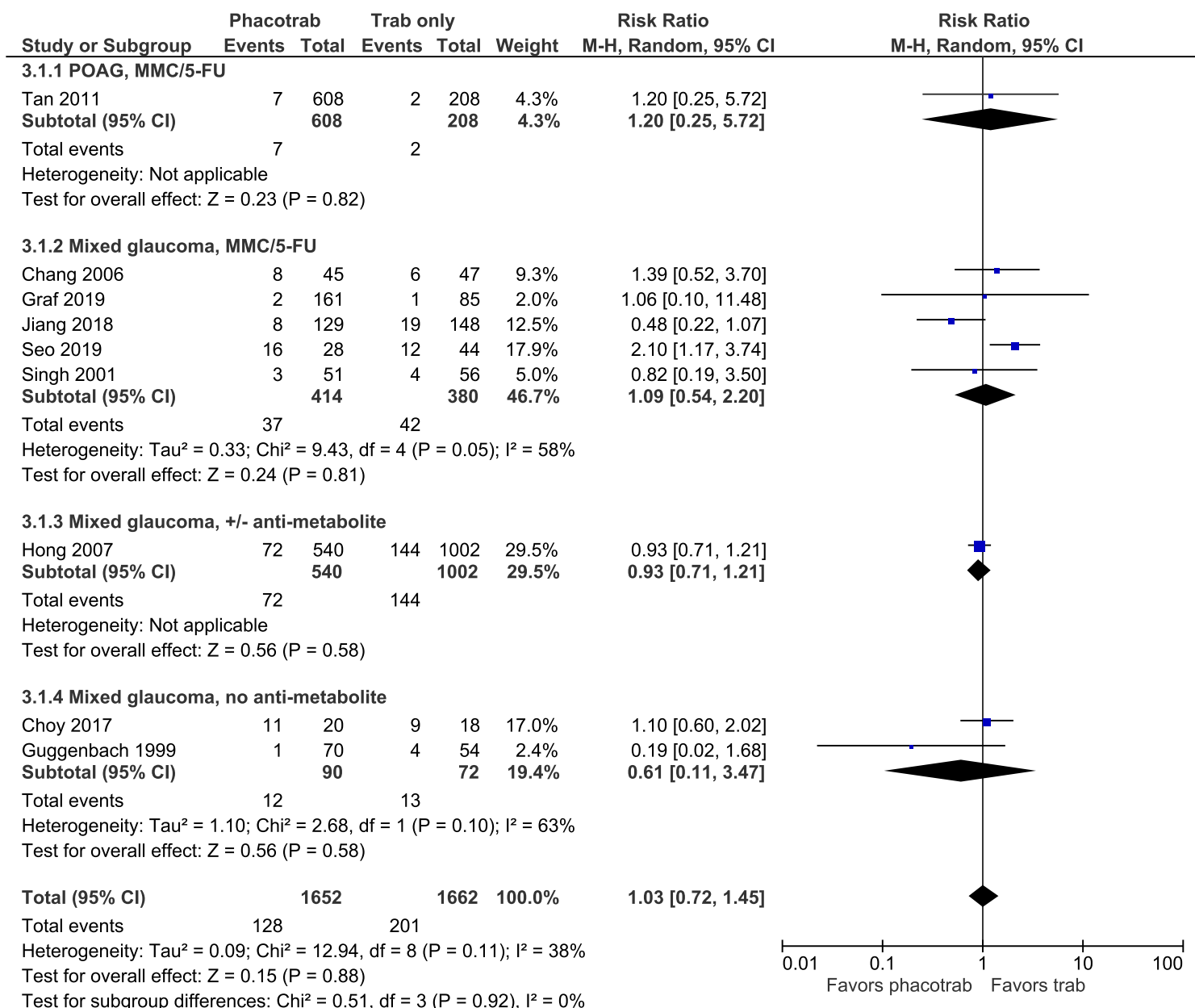

Supplement: Supplementary Materials — Supplementary File 1: list of excluded studies. Supplementary File 2: a review of evidence quality. Supplementary Figure S1: forest plot of the risk of complications after phacotrabeculectomy versus phacoemulsification 3–6 months after trabeculectomy. Supplementary Figure S2: forest plot of the visual field after phacotrabeculectomy versus trabeculectomy only. Supplementary Figure S3: forest plot of the risk of needling or revision after phacotrabeculectomy versus trabeculectomy only. Supplementary Figure S4: forest plot of the complete success after phacotrabeculectomy versus trabeculectomy only. Supplementary Figure S5: forest plot of the qualified success after phacotrabeculectomy versus trabeculectomy only. Supplementary Figure S6: forest plot of the surgical failure after phacotrabeculectomy versus trabeculectomy only. Supplementary Figure S7: forest plot of the difference in a number of antiglaucomatous medications after phacotrabeculectomy versus trabeculectomy only. [file 6682534.f1.zip › S3.pdf]

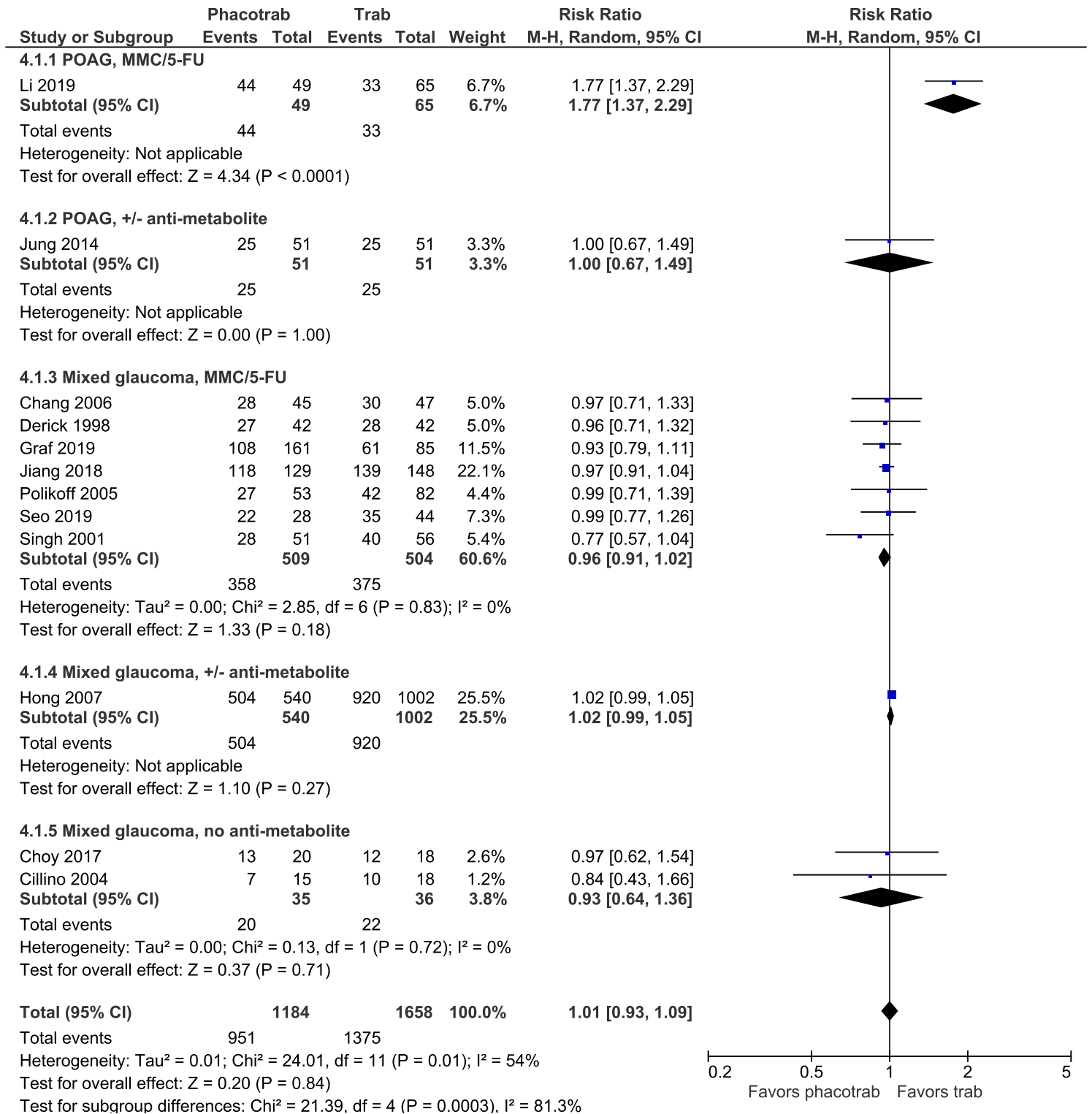

Supplement: Supplementary Materials — Supplementary File 1: list of excluded studies. Supplementary File 2: a review of evidence quality. Supplementary Figure S1: forest plot of the risk of complications after phacotrabeculectomy versus phacoemulsification 3–6 months after trabeculectomy. Supplementary Figure S2: forest plot of the visual field after phacotrabeculectomy versus trabeculectomy only. Supplementary Figure S3: forest plot of the risk of needling or revision after phacotrabeculectomy versus trabeculectomy only. Supplementary Figure S4: forest plot of the complete success after phacotrabeculectomy versus trabeculectomy only. Supplementary Figure S5: forest plot of the qualified success after phacotrabeculectomy versus trabeculectomy only. Supplementary Figure S6: forest plot of the surgical failure after phacotrabeculectomy versus trabeculectomy only. Supplementary Figure S7: forest plot of the difference in a number of antiglaucomatous medications after phacotrabeculectomy versus trabeculectomy only. [file 6682534.f1.zip › S4.pdf]

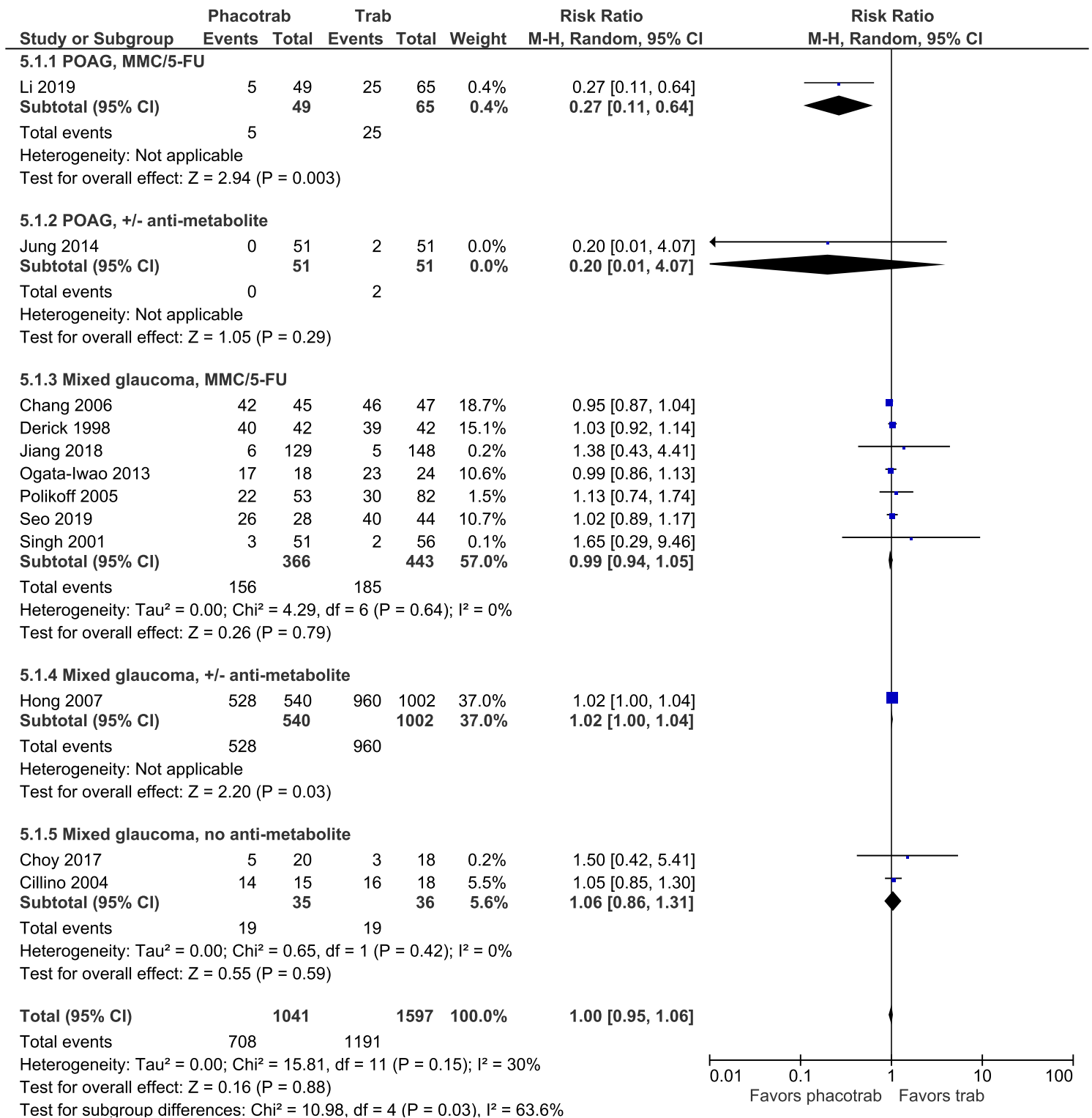

Supplement: Supplementary Materials — Supplementary File 1: list of excluded studies. Supplementary File 2: a review of evidence quality. Supplementary Figure S1: forest plot of the risk of complications after phacotrabeculectomy versus phacoemulsification 3–6 months after trabeculectomy. Supplementary Figure S2: forest plot of the visual field after phacotrabeculectomy versus trabeculectomy only. Supplementary Figure S3: forest plot of the risk of needling or revision after phacotrabeculectomy versus trabeculectomy only. Supplementary Figure S4: forest plot of the complete success after phacotrabeculectomy versus trabeculectomy only. Supplementary Figure S5: forest plot of the qualified success after phacotrabeculectomy versus trabeculectomy only. Supplementary Figure S6: forest plot of the surgical failure after phacotrabeculectomy versus trabeculectomy only. Supplementary Figure S7: forest plot of the difference in a number of antiglaucomatous medications after phacotrabeculectomy versus trabeculectomy only. [file 6682534.f1.zip › S5.pdf]

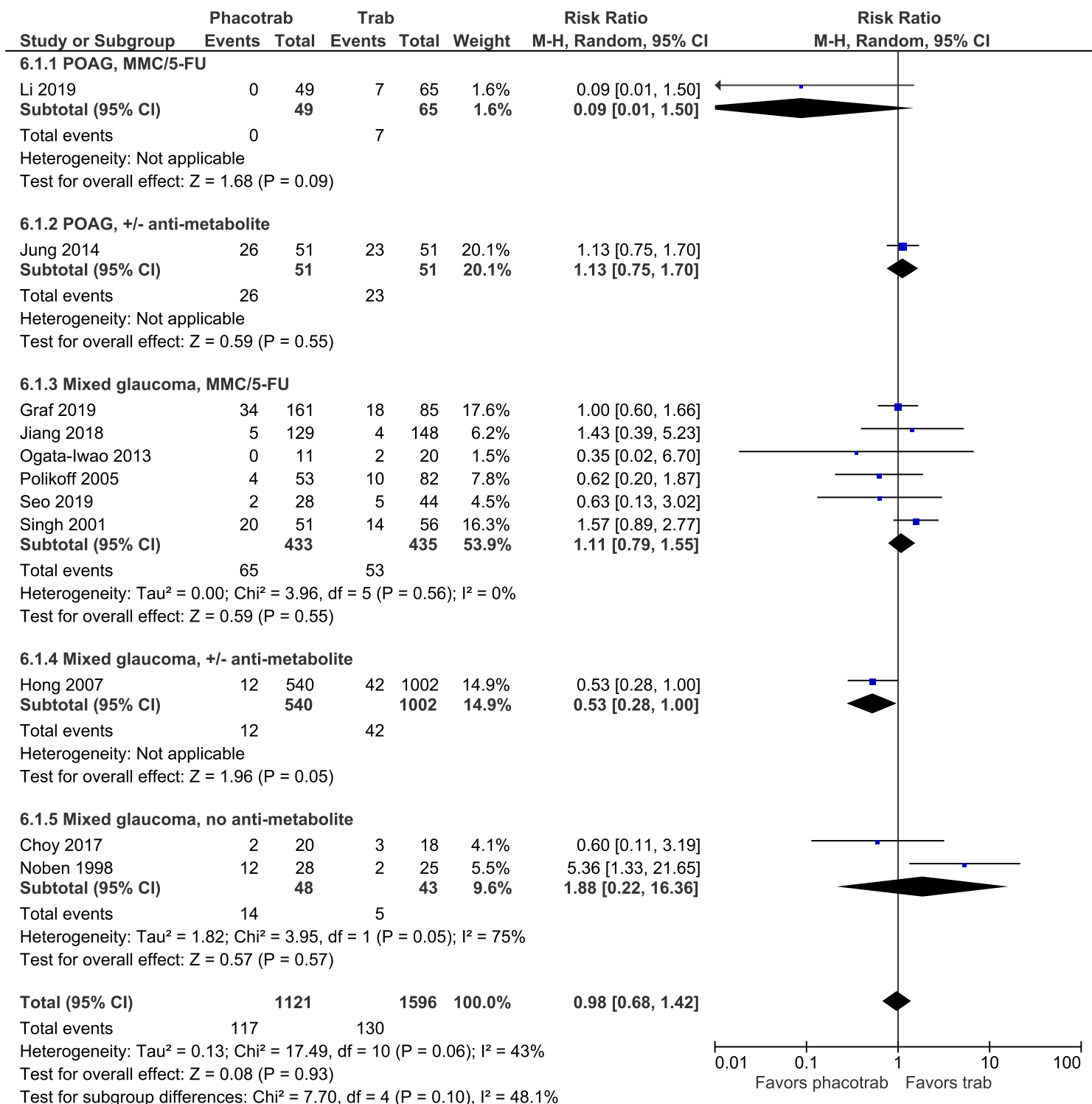

Supplement: Supplementary Materials — Supplementary File 1: list of excluded studies. Supplementary File 2: a review of evidence quality. Supplementary Figure S1: forest plot of the risk of complications after phacotrabeculectomy versus phacoemulsification 3–6 months after trabeculectomy. Supplementary Figure S2: forest plot of the visual field after phacotrabeculectomy versus trabeculectomy only. Supplementary Figure S3: forest plot of the risk of needling or revision after phacotrabeculectomy versus trabeculectomy only. Supplementary Figure S4: forest plot of the complete success after phacotrabeculectomy versus trabeculectomy only. Supplementary Figure S5: forest plot of the qualified success after phacotrabeculectomy versus trabeculectomy only. Supplementary Figure S6: forest plot of the surgical failure after phacotrabeculectomy versus trabeculectomy only. Supplementary Figure S7: forest plot of the difference in a number of antiglaucomatous medications after phacotrabeculectomy versus trabeculectomy only. [file 6682534.f1.zip › S6.pdf]

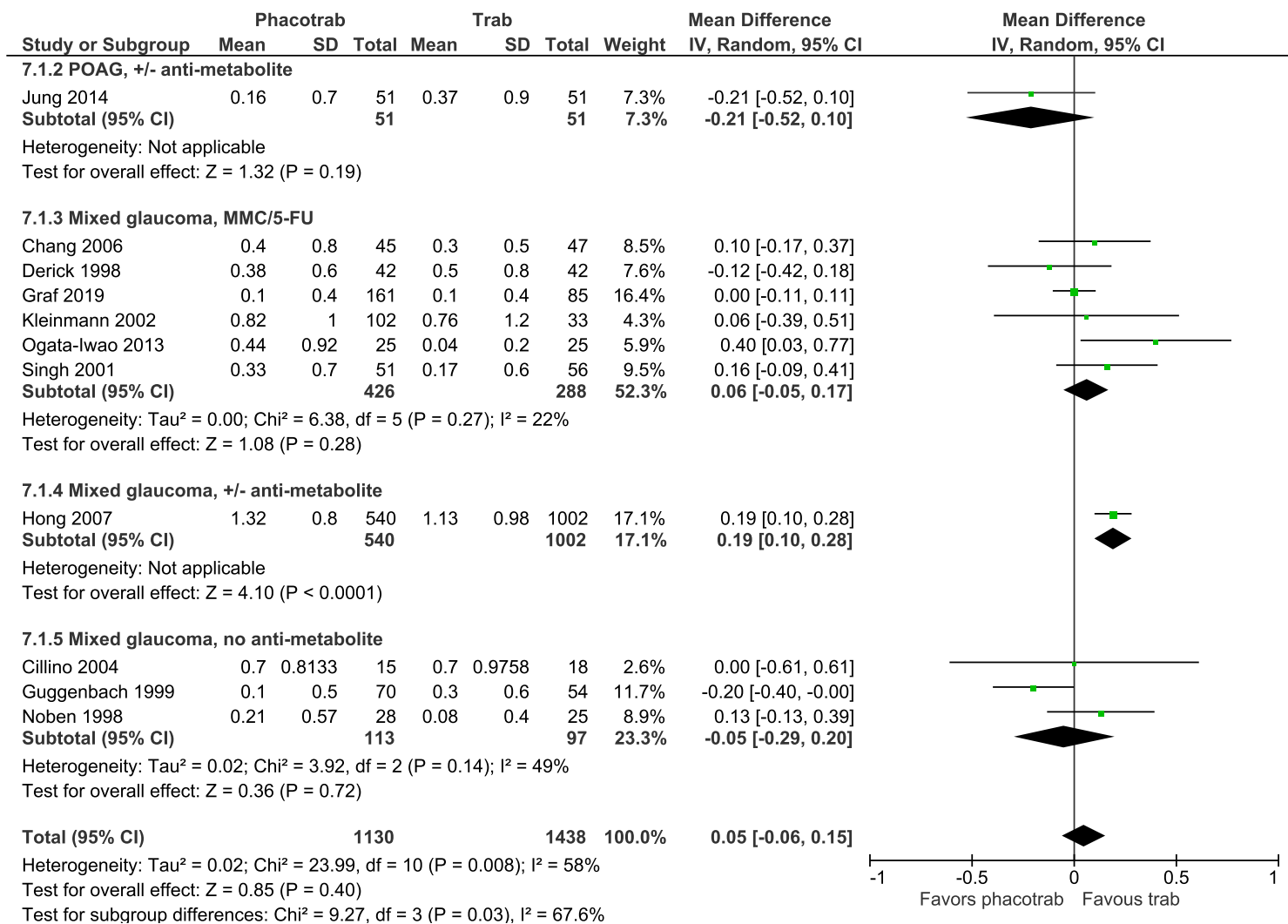

Supplement: Supplementary Materials — Supplementary File 1: list of excluded studies. Supplementary File 2: a review of evidence quality. Supplementary Figure S1: forest plot of the risk of complications after phacotrabeculectomy versus phacoemulsification 3–6 months after trabeculectomy. Supplementary Figure S2: forest plot of the visual field after phacotrabeculectomy versus trabeculectomy only. Supplementary Figure S3: forest plot of the risk of needling or revision after phacotrabeculectomy versus trabeculectomy only. Supplementary Figure S4: forest plot of the complete success after phacotrabeculectomy versus trabeculectomy only. Supplementary Figure S5: forest plot of the qualified success after phacotrabeculectomy versus trabeculectomy only. Supplementary Figure S6: forest plot of the surgical failure after phacotrabeculectomy versus trabeculectomy only. Supplementary Figure S7: forest plot of the difference in a number of antiglaucomatous medications after phacotrabeculectomy versus trabeculectomy only. [file 6682534.f1.zip › S7.pdf]
